# CRISPR/Cas9 editing of APP C-terminus attenuates β-cleavage and promotes α-cleavage

**DOI:** 10.1038/s41467-018-07971-8

**Published:** 2019-01-03

**Authors:** Jichao Sun, Jared Carlson-Stevermer, Utpal Das, Minjie Shen, Marion Delenclos, Amanda M. Snead, So Yeon Koo, Lina Wang, Dianhua Qiao, Jonathan Loi, Andrew J. Petersen, Michael Stockton, Anita Bhattacharyya, Mathew V. Jones, Xinyu Zhao, Pamela J. McLean, Andrew A. Sproul, Krishanu Saha, Subhojit Roy

**Affiliations:** 1 Department of Pathology and Laboratory Medicine, University of Wisconsin-Madison, 1111 Highland Avenue, Madison, WI 53705 USA; 2 Department of Biomedical Engineering, University of Wisconsin-Madison, 1550 Engineering Drive, Madison, WI 53706 USA; 3 Wisconsin Institute for Discovery, University of Wisconsin-Madison, 330 N. Orchard, Madison, WI 53715 USA; 4 Department of Neuroscience, University of California, San Diego, 9500 Gilman Drive, La Jolla, CA 92093 USA; 5 Waisman Center, University of Wisconsin-Madison, 1500 Highland Ave, Madison, WI 53705 USA; 6 Department of Neuroscience, Mayo Clinic Jacksonville, 4500 San Pablo Rd, Jacksonville, FL 32224 USA; 7 Taub Institute for Research on Alzheimer’s and the Aging Brain, Columbia University Medical Center, 630W 168th St, New York, NY 10032 USA; 8 Department of Neuroscience, University of Wisconsin-Madison, 1111 Highland Avenue, Madison, WI 53705 USA; 9 Department of Pathology and Cell Biology, Columbia University Medical Center, 630W 168th St, New York, NY 10032 USA

## Abstract

CRISPR/Cas9 guided gene-editing is a potential therapeutic tool, however application to neurodegenerative disease models has been limited. Moreover, conventional mutation correction by gene-editing would only be relevant for the small fraction of neurodegenerative cases that are inherited. Here we introduce a CRISPR/Cas9-based strategy in cell and animal models to edit endogenous amyloid precursor protein (APP) at the extreme C-terminus and reciprocally manipulate the amyloid pathway, attenuating APP-β-cleavage and Aβ production, while up-regulating neuroprotective APP-α-cleavage. APP N-terminus and compensatory APP-homologues remain intact, with no apparent effects on neurophysiology in vitro. Robust APP-editing is seen in human iPSC-derived neurons and mouse brains with no detectable off-target effects. Our strategy likely works by limiting APP and BACE-1 approximation, and we also delineate mechanistic events that abrogates APP/BACE-1 convergence in this setting. Our work offers conceptual proof for a selective APP silencing strategy.

## Abstract (web summary)

Gene editing strategies are typically designed to correct mutant genes, but most neurodegenerative diseases are sporadic. Here the authors describe a strategy to selectively edit the C-terminus of APP and attenuate amyloid-β production, while upregulating neuroprotective α-cleavage.

## Introduction

CRISPR/Cas9-guided gene editing is emerging as a promising tool to disrupt the expression of disease-causing genes or edit pathogenic mutations^1^. Recent proof-of-principle studies have highlighted the feasibility of this powerful technique as interventional tools for neurodegenerative diseases^2–5^. However, current approaches relying on canonical gene-deletion or mutation-correction using CRISPR-technology are limited in practicability and scope. First, elimination of entire genes would almost certainly have deleterious effects on physiology, since most of these genes have normal roles as well. Secondly, strategies aimed at correcting point-mutations would only be applicable to the small fraction of neurodegenerative diseases that are inherited (typically < 10% of cases). Moreover, a different editing-approach would be required for each gene mutation—further complicating intervention—and fresh ideas are needed to help realize the potential of gene-editing in sporadic disease.

A common theme in neurodegenerative diseases is that proteins normally present in the brain (APP, tau, α-synuclein, TDP-43, etc.) acquire toxic properties—or trigger pathologic cascades—that ultimately lead to synaptic loss and neurodegeneration. Our broad idea is to rationally edit small segments of endogenous proteins known to play key roles in the progression of disease, with the ultimate goal of attenuating their pathologic activity. As endogenous proteins expectedly play physiologic roles, it is also important to conserve their normal function, as far as possible. Here we show conceptual proof of this selective silencing approach for APP. APP is a single-pass transmembrane protein, cleaved by the enzymes β-secretases and γ-secretases to ultimately generate Aβ–a neuropathologic hallmark of AD. APP cleavage by the enzyme β-secretase BACE-1 is the rate limiting step in this amyloidogenic pathway. Alternatively, APP is cleaved by α-secretases—the non-amyloidogenic pathway—that is thought to be neuroprotective because it precludes β-cleavage of APP^6,7^; and studies have highlighted neuroprotective effects of APP-α-cleavage products in vivo^8,9^.

We recently developed a Bi-molecular fluorescence complementation (BifC) assay to visualize the physical approximation of APP and BACE-1 in neurons^10^. As a control for assay-validation, we found that a C-terminus deletion also abrogated APP/BACE-1 complementation^10^; in line with previous studies showing that deletions/mutations of the APP C-terminus can attenuate Aβ production^11–13^. Thus we had the idea of using CRISPR/Cas9-mediated truncation of native APP to attenuate APP-β-cleavage and Aβ production in AD. Using CRISPR-tools, cell/molecular biology, live imaging, deep sequencing, electrophysiology and in vivo animal studies, here we highlight a strategy to favorably manipulate the amyloid pathway by gene editing.

## Results

### CRISPR/Cas9 editing of APP C-terminus

**Fig. 1:**
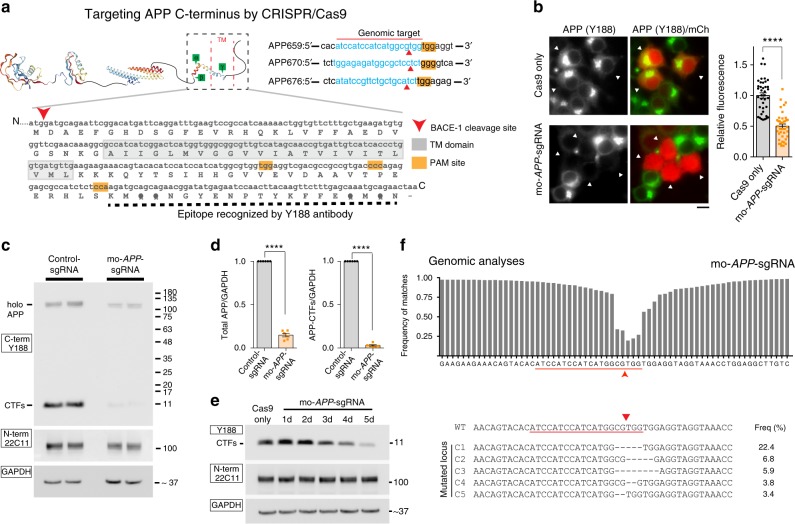
Manipulation of the amyloid pathway by CRISPR/Cas9 editing. **a** Schematic and C-terminal sequence of mouse APP showing PAM sites (yellow) and genomic targets for the three *APP*-sgRNAs (*APP*659-sgRNA used henceforth and referred to as ‘*APP*-sgRNA’ – see text). Note that the C-terminal antibody Y188 recognizes the last 20 aa. of APP (black dashed line). **b** Neuro2a cells were transfected with mo-*APP*-sgRNA and Cas9 (or Cas9 only), and immunostained with the Y188 antibody (after 5 days; mCherry labels transfected cells). Note decreased APP (Y188) fluorescence, indicating APP editing (quantified on right, mean ± SEM of 39 cells from two independent experiments per condition). Scale bar 10 μm. **c**, **d** Neuro2a cells were transduced by lentiviral vectors carrying mo-*APP*-sgRNA and Cas9 (or non-targeting control-sgRNA/Cas9 as control) and immunoblotted with Y188 and 22C11 antibodies (latter recognizes APP N-terminus). A gamma secretase inhibitor (GSI) was added to allow detection of accumulated APP CTF’s (see methods, GAPDH used as loading controls). Note attenuated signal with the Y188 antibody in mo-*APP*-sgRNA treated samples, but no change in 22C11 signal. Blots quantified in **d**, mean ± SEM of six independent experiments. **e** Time course of APP-editing in neuro2a cells. Cells were transfected with a vector carrying mo-*APP*-sgRNA and Cas9, and APP-CTFs were analyzed by Western blotting (in the presence of GSI). **f** Deep sequencing of APP C-terminus in neuro2a cells. Top: Frequency of base-pair matches between  CRISPR-edited and WT mouse sequence. Red underline marks the sgRNA target sequence and arrowhead denotes predicted cut-site. Note extensive mismatch around predicted cut-site, indicating robust editing. Bottom: Major mutated *APP* loci resulting from sgRNA-editing, and their frequencies. For all panels, significance determined with two-tailed *t*-test, ****p < 0.0001. Source data are provided as a Source Data file

The CRISPR/Cas9 system consists of a Cas9 nuclease enzyme that generates double-stranded breaks in DNA, and a custom-designed single guide-RNA (sgRNA) that targets the Cas9 to specific sites in the host genomic DNA. Typically, the synthetic sgRNAs are complementary to stretches of genomic DNA containing 3-nt PAM (protospacer adjacent motif) and flanking 20-nt sequences. Since subsequent repair after DNA-breaks is naturally error-prone, insertions and deletions (indels) are generated at the cut-sites, leading to disruption of the translational reading frame and effectively truncated proteins (reviewed in^14^). We identified three PAM sites at the APP C-terminus that are conserved in both human and mouse, and synthesized sgRNAs targeting these regions (Fig. 1a). To compare the editing efficiency of these sgRNAs, we engineered a stable H4 neuroglioma cell line expressing single copies of APP:VN and BACE-1:VC (APP/BACE^single_copy^), where editing efficiency of a given sgRNA could be determined as a simple on/off fluorescence readout and the effect of APP truncation could be assessed by evaluating secreted Aβ (for details, see Supplementary Fig. 1a, b and methods). The *APP*-sgRNA predicted to cut human APP at the 659 aa. (amino acid) position was the most efficient—both in editing APP, as well as in attenuating Aβ—and also led to minimal indels (Supplementary Fig. 1c–e and Supplementary Table 1). Accordingly, we used the *APP*659-sgRNA for further characterization (henceforth called ‘mo-*APP*-sgRNA’ or ‘hu-*APP*-sgRNA’ representing mouse and human specific sequences).

**Fig. 2:**
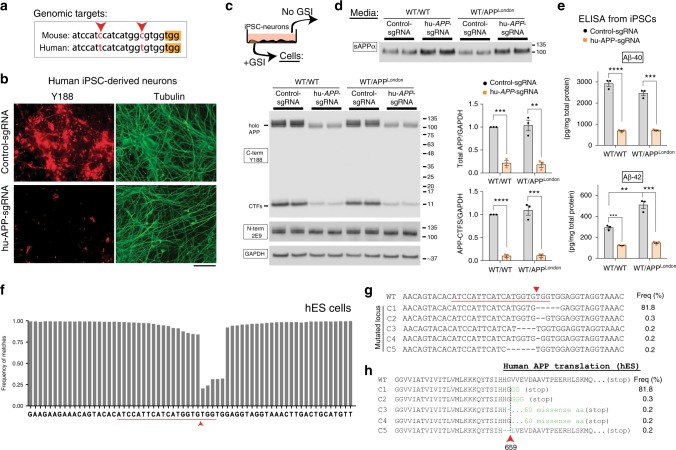
Gene editing of APP C-terminus and effects on APP processing in human cells. **a** Comparison of mouse and human *APP*-sgRNA targeting sequences (red arrowheads indicate differences; yellow bar denotes the PAM site). **b** Human iPSC-derived NPCs were transduced by lentiviral vectors carrying hu-*APP*-sgRNA and Cas9 (or non-targeting control-sgRNA/Cas9 as control) and differentiated into neurons. After 3 weeks of differentiation, cells were immunostained with the Y188 and Tuj1 (tubulin) antibodies. Note decreased APP (Y188) fluorescence, indicating APP editing. Scale bar 50 μm. **c** The iPSC-derived neurons above (or isogenic APPV717I London-mutant knock-in iPSC-neurons) were transduced and differentiated as above and immunoblotted with C- and N-terminus antibodies (GSI was added to allow detection of accumulated APP CTFs). Note attenuation of APP signal with Y188 after hu-*APP*-sgRNA treatment in both wild type and isogenic APP^London^ iPSCs (quantified on right, mean ± SEM of three independent experiments). **d** Media from the iPSC-derived neurons above was immunoblotted for secreted sAPPα (6E10 antibody). Note increased sAPPα in sgRNA-treated samples, indicating upregulation of the non-amyloidogenic pathway. **e** ELISA of media from iPSC derived neurons. Note decreased Aβ in the sgRNA-treated samples (mean ± SEM of three independent experiments). **f** Deep sequencing of APP C-terminus in human ESCs. Red underline marks the sgRNA target sequence and arrowhead denotes predicted cut-site. Note extensive mismatch around predicted cut-site, indicating robust editing. **g** Major mutated *APP*-loci resulting from CRISPR editing, and their frequencies. **h** Predicted APP translational products (post-editing) for the major mutant alleles observed in deep sequencing. Note that after editing, APP is translated up-to amino acid 659 (red arrowheads; similar results were seen in HEK cells, see Supplementary Fig. 1e). For all panels, significance determined with two-tailed *t*-test, **p < 0.01, ***p < 0.001, ****p < 0.0001. Source data are provided as a Source Data file

The TGG PAM and preceding 20-nt genomic target sequence recognized by the mo-*APP*-sgRNA is shown in Fig. 1a (top right); and Fig. 1b–f shows gene editing by this sgRNA in mouse cells. Note that upon editing, the Y188 antibody—recognizing the last 20 aa. of APP—would not be able to identify the resultant translational product. Robust editing of endogenous APP was seen in mouse neuroblastoma cells, as determined by attenuation of immunofluorescence with the Y188 antibody (Fig. 1b), and decreased Y188-signal in western blots (Fig. 1c, d; Fig. 1e shows time-course of editing). Note that the edited APP is recognized by antibodies to the N-terminus, indicating selective editing of the C-terminus by the mo-*APP*-sgRNA (Fig. 1c, e). However, the N-terminus antibody was unable to detect APP when the entire gene was deleted (Supplementary Fig. 2a and Supplementary Table 1). Similar results were obtained with other sgRNAs targeting APP C-terminus and other C-terminus and N-terminus APP antibodies (Supplementary Fig. 2b, c and Supplementary Table 1). Genomic deep-sequencing confirmed efficient editing of mouse APP at the expected loci, APP-659 (Fig. 1f). Post-editing translational products show that the last 36 aa. are effectively truncated by mo-*APP*-sgRNA (Supplementary Fig. 2d). Though the TGG PAM at this site is conserved in both mouse and human *APP*, and the upstream sgRNA-targeting sequences only differ by two nucleotides (Fig. 2a, arrowheads); the mo-*APP*-sgRNA was unable to edit human APP. However, a sgRNA specific to the human *APP* targeting sequence robustly edited APP in HEK293 (Supplementary Fig. 3a–c), as well as in human embryonic stem cells (Supplementary Fig. 3d, e). CRISPR editing of APP did not alter the steady-state levels of holo-APP (note data throughout with multiple N-terminus antibodies in various cell lines).

### Effect of APP C-terminus editing on β/α pathway

Next, we examined APP editing in human iPSC-derived neurons. As shown in Fig. 2b, immunostaining with the Y188 antibody was attenuated in iPSC-neurons transduced by the hu-*APP*-sgRNA. To examine effects of APP editing in an “AD-like setting”, we also tested the hu-*APP*-sgRNA in a heterozygous knock-in iPSC line carrying the most common familial AD (*APP*) mutation (APPV717I, also called the ‘London mutation’; see methods for details of iPSC line). Both cell-lysates and supernatants were examined, to look for cellular and secreted APP products (see schematic in Fig. 2c). Immunoblotting with the Y188 antibody confirmed robust—and C-terminus selective—APP editing in both WT and APP-London iPSC lines (Fig. 2c). Examination of supernatants revealed that interestingly, APP-editing also led to increased sAPPα in both WT and London lines (Fig. 2d); suggesting upregulation of the neuroprotective α-cleavage pathway. ELISAs and western blot showed attenuated secretion of Aβ40/42 (Fig. 2e) and sAPPβ (Supplementary Fig. 3f), confirming inhibition of the amyloidogenic pathway in these neurons. Genomic deep sequencing showed efficient editing of human APP by the sgRNA, with truncation of the last 36 aa. in human embryonic stem cells (Fig. 2f–h).

The data from iPSC-neurons suggest that the *APP*-sgRNA has reciprocal effects on APP β- and α-cleavage. To validate this in a more controlled setting, we tested the effects of APP editing in the H4 APP/BACE^single_copy^ cell line, where APP-cleavage is tightly regulated. In line with the data from iPSC-neurons, the hu-*APP*-sgRNA had reciprocal effects on APP β-cleavage and α-cleavage in APP/BACE^single_copy^ cells as well, confirming that our editing strategy has reciprocal effects on β/α cleavage (Supplementary Fig. 3g). Further experiments using an APP-C99 construct (wild-type and truncated construct mimicking the CRISPR-product, APP-659) precludes an effect of the sgRNA on APP-γ-cleavage (Supplementary Fig. 4), indicating that our editing strategy is selectively affecting APP β-cleavage. Collectively, the available data strongly suggest that our gene editing strategy targeting the APP C-terminus is favorably manipulating the amyloid pathway by attenuating APP β-cleavage, while reciprocally up-regulating protective α-cleavage.

### Off-target analysis and effects on neurophysiology

Off-target effects of CRISPR/Cas9, due to unwanted editing of DNA-stretches resembling the targeted region, are a concern. Towards this, we asked if our mouse and human *APP*-sgRNA were able to edit the top five computationally predicted off-target sites (Supplementary Fig. 5a; also see Supplementary Table 2). No editing was seen using T7 endonuclease assays (Supplementary Fig. 5b–e). Though *APP* null mice are viable, there is compensation by the two APP homologues APLP1 and 2 that undergo similar processing as APP^15,16^. *APLP1* and *APLP2* were not amongst the top 50 predicted off-target sites, as their corresponding sgRNA-target sites were substantially different from *APP* (see sequences in Supplementary Fig. 5f). For further assurance that our sgRNA was not editing the APP homologues, we performed specific off-target TIDE (Tracking of Indels by DEcomposition) analyses^17^ on cells carrying the sgRNA. As shown in Supplementary Fig. 5g, TIDE analyses showed no editing of *APLP 1/2* by the sgRNA.

**Fig. 3:**
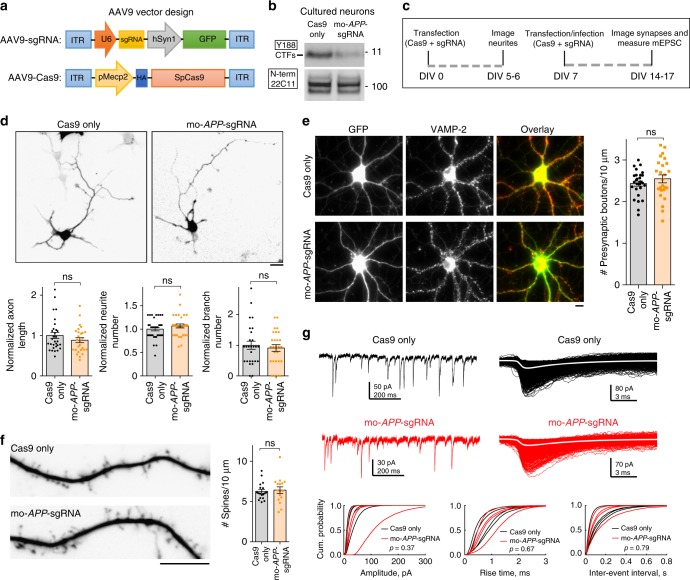
Effect of APP C-terminus editing on neuronal physiology. **a** AAV9-sgRNA and AAV9-Cas9 expression vectors. Note that the sgRNA vector co-expresses GFP and the Cas9 is tagged to HA, for identification of transduced neurons. **b** Cultured hippocampal neurons were transduced with AAV9s carrying mo-*APP*-sgRNA/Cas9 (or Cas9 only) and immunoblotted with the Y188 and 22C11 antibodies (with GSI). Note attenuation of CTFs by the mo-*APP*-sgRNA. **c** Neurons were transfected (at the time of plating) with *APP* CRISPR. Neuritic/axon outgrowth was analyzed after 5-6 days. Neurons were transfected or infected at DIV7 with *APP* CRISPR, and synapse structure/function was analyzed after 14–17 days. **d** Top: Representative images of neurons transfected with the mo-*APP*-sgRNA/Cas9 (or Cas9 only). Bottom: Quantifications of axon length and number of neurites/branches; note that there was no significant difference (mean ± SEM; axon length: 30 cells for control and 27 cells for mo-*APP*-sgRNA from two independent experiments, *p* = 0.2462; neurite number: 35 cells for control and 31 cells for mo-*APP*-sgRNA from two independent experiments, *p* = 0.2289; branch number: 27 cells for both conditions from two independent experiments, *p* = 0.6008 by two-tailed *t*-test*;* ns = non-significant). Scale bar 20 μm. **e** Neurons were infected with mo-*APP*-sgRNA/Cas9 (or Cas9 only), and fixed/stained with the presynaptic marker VAMP2. Note that the presynaptic density (VAMP2 puncta) was similar in both groups (mean ± SEM of VAMP2 staining along 27 dendrites for control and 25 dendrites for mo-*APP*-sgRNA from two independent experiments, *p* = 0.3132 by two-tailed *t*-test). Scale bar 10 μm. **f** Neurons were transfected with mo-*APP*-sgRNA/Cas9 (or Cas9 only as controls). Spine density in both groups was similar (mean ± SEM of 18 dendrites for control and 16 dendrites for mo-*APP*-sgRNA from two independent experiments, *p* = 0.7456 by two-tailed *t*-test). Scale bar 10 μm. **g** Miniature excitatory postsynaptic currents (mEPSC) were recorded from neurons infected with AAV9-*APP*-sgRNA/Cas9. Top: Representative mEPSC traces in control and mo-*APP*-sgRNA transduced neurons. Corresponding alignments of mEPSCs with average (white traces) are shown on right. Bottom: Cumulative histograms of mEPSC amplitude, 20–80% rise-time and inter-event interval in mo-*APP*-sgRNA/Cas9 and the Cas9-only infected neurons (note no significant differences). Source data are provided as a Source Data file

APP has known physiologic roles in axon growth and signaling^18^. As noted above, the N-terminus of APP—thought to play roles in axon growth and differentiation—is entirely preserved in our setting. The C-terminal APP intracellular domain (AICD) has been implicated in gene transcription, though the effect appears to be both physiologic and pathologic^19,20^. To examine potential deleterious effects of editing the extreme C-terminus of APP, we turned to cultured hippocampal neurons where various parameters like neurite outgrowth and synaptic structure/function can be confidently evaluated. To study pre-synapse structure and neuronal activity, we generated AAV9 viruses carrying the mo-*APP*-sgRNA and Cas9, tagged with GFP and HA, respectively (see vector design in Fig. 3a) that transduced almost all cultured neurons (Fig. 3b; 89.77 ± 2.27% neurons infected with mo-*APP*-sgRNA AAV and 88.03 ± 1.42% neurons infected with control AAV, mean ± SEM from three independent experiments). In blinded analyses, we found no significant effect of the mo-*APP*-sgRNA on neurite outgrowth, axon-length, synaptic organization, or neuronal activity (Fig. 3c–g). We reason that the lack of deleterious effects upon editing is likely because: (1) most of the APP molecule remains intact after editing; (2) the APP homologues APLP1/2—that undergo similar processing as APP, generate CTFs, and are known to compensate for APP function—remain unedited; and (3) APP-cleavage is not entirely blocked by our approach.

### APP editing in vivo and mechanism of APP β/α manipulation

**Fig. 4:**
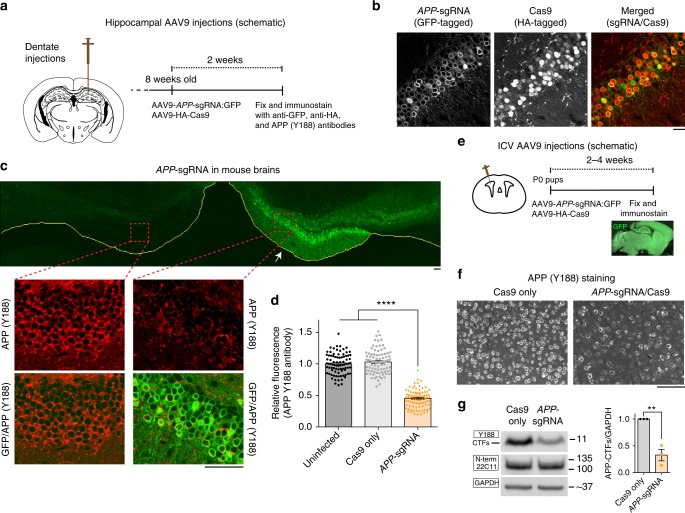
Gene editing of APP C-terminus in vivo. **a** AAV9-sgRNA and AAV9-Cas9 were stereotactically co-injected into dentate gyrus of 8-week old mouse brains (bottom). Two weeks after viral delivery, brains were perfused, fixed, and immunostained with anti-GFP, anti-HA and anti-APP(Y188) antibodies. **b** Co-expression of AAV9-sgRNA-GFP and AAV9-HA-Cas9 in the dentate gyrus. Note that majority of neurons are positive for both GFP and HA (~87% of the cells were positive for both; sampling from 3 brains). Scale bar 20 μm. **c**, **d** Coronal section of a mouse hippocampi injected on one side (marked by arrow) with the AAV viruses as described above. Note attenuated Y188 staining of neurons on the injected side, indicating APP-editing. The image of mouse hippocampus injected with Cas9 only is not shown. Fluorescence quantified in **d**, mean ± SEM, data from three brains. One-way ANOVA: *p* < 0.0001. Tukey’s multiple comparisons: *p* = 0.4525 (Un-injected vs. Cas9 only); *p* < 0.0001 (Un-injected vs. *APP*-sgRNA); *p* < 0.0001 (Cas9 only vs. *APP*-sgRNA). Scale bars 50 μm. **e** Intracerebroventrical injection of the AAV9 viruses into P0 pups. Note widespread delivery of sgRNA into brain, as evident by GFP fluorescence. Scale bar 100 μm. **f** Brain sections from above were immunostained with the Y188 antibody. Note attenuated Y188 staining in the *APP*-sgRNA/Cas9 transduced sample, suggesting APP-editing. **g** Western blots of the brains from **e**. Note decreased expression of CTFs in the *APP*-sgRNA/Cas9 transduced brains; blots quantified on right (mean ± SEM of three independent experiments, **p < 0.01 by two-tailed *t*-test). Source data are provided as a Source Data file

Next we asked if the *APP*-sgRNA could edit endogenous APP in mouse brains. Injection of the AAV9s into mouse hippocampi (Fig. 4a) led to efficient transduction of both sgRNA and Cas9 in dentate neurons (86.87 ± 2.83% neurons carrying the sgRNA also had Cas9, mean ± SEM from three independent experiments; see representative images in Fig. 4b). Immunostaining of transduced neurons with the APP Y188 antibody showed attenuated staining, suggesting editing of endogenous APP in vivo (Fig. 4c, d). To achieve a more widespread expression of the sgRNA and Cas9 in mouse brains—and also evaluate editing by biochemistry—we injected the viruses into the ventricles of neonatal (P0) mice and examined the brain after 2–4 weeks (Fig. 4e). Previous studies have shown that when AAVs are injected into the ventricles of neonatal mice, there is widespread delivery of transgenes into the brain—also called somatic transgenesis^21,22^. Indeed, APP Y188 immunostaining was attenuated in cortical regions (Fig. 4f) and immunoblotting with the Y188 antibody also showed a decreased signal (Fig. 4g); indicating that the mo-*APP*-sgRNA can edit APP in vivo.

**Fig. 5:**
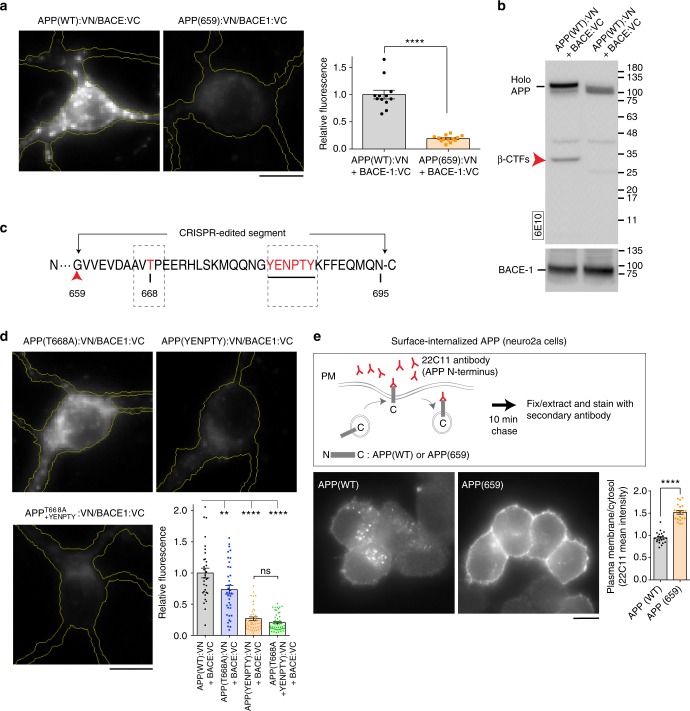
Mechanistic details of CRISPR-guided APP editing. **a** APP/BACE-1 interaction—as evaluated by fluorescence complementation in cultured hippocampal neurons—was attenuated in neurons transfected with an APP C-terminus truncation mimicking the post-edited translational product (APP659:VN; quantified below, mean ± SEM of 12 cells for APP(WT) and 13 cells for APP(659) from two independent experiments, *p* < 0.0001 by two-tailed *t*-test). Scale bar 10 μm. **b** APP β-cleavage is also attenuated in cells transfected with APP659. HEK cells were co-transfected with APPWT (or APP659) tagged to VN, and BACE-1:VC; and immunoblotted with the 6E10 antibody. Note decreased β-CTFs in cells carrying the truncated APP plasmid. **c** Schematic showing the CRISPR-edited C-terminus portion of APP. Note that the threonine at 668 position, and the endocytic YENPTY motif (dashed boxes) are thought to play roles in Aβ production (see text). **d** APP/BACE-1 interaction—as evaluated by fluorescence complementation in cultured hippocampal neurons—was most markedly attenuated in neurons transfected with mutant YENPTY (mean ± SEM of 32 cells for APP(WT), 37 cells for APP(T668A), 45 cells for APP(YENPTY) and 49 cells for APP(T668A + YENPTY) from two independent experiments). One-way ANOVA: *p* < 0.0001. Tukey’s multiple comparisons: *p* = 0.0022 (APP vs. APP^T668A^); *p* < 0.0001 (APP vs. APP^YENPTY^); *p* < 0.0001 (APP vs. APP^T668A+YENPTY^); *p* < 0.0001 (APP^T668A^ vs. APP^YENPTY^); *p* < 0.0001 (APP^T668A^ vs. APP^T668A+YENPTY^); *p* = 0.7568 (APP^YENPTY^ vs. APP^T668A+YENPTY^). Scale bar 10 μm. **e** Strategy of APP internalization assay. Neuro 2a cells are transfected with APP:GFP or APP659:GFP. After incubation with anti N-terminal APP antibody (22C11) for 10 min, the cells were fixed and stained with secondary antibody to visualize the cell surface and internalized APP. Note the cell surface accumulation and decreased internalization of APP659 (mean ± SEM of 21 cells from two independent experiments, *p* < 0.0001 by two-tailed *t*-test). Scale bar 10 μm. Source data are provided as a Source Data file

To determine the mechanism by which the *APP*-sgRNA manipulates the amyloid pathway, we used a “CRISPR-mimic” truncated APP construct (APP-659) that is the major post-editing translational product in both mouse and human cells (see Fig.2h, Supplementary Fig. 1e, and Supplementary Fig. 2d). Using our BifC assay^10^, we first asked if the CRISPR-mimic APP-659 interacted with BACE-1. APP-659/BACE-1 approximation was greatly attenuated in cultured neurons (Fig. 5a), along with a decrease in β-CTF generation (Fig. 5b). Next we visualized axonal and dendritic transport of APP-WT and APP-659. Although there were minor changes (Supplementary Fig. 6 and Supplementary Table 3), it seems unlikely that such small transport perturbations would lead to the dramatic attenuation of β-cleavage and Aβ-production seen in our experiments.

The CRISPR-edited segment of APP contains the residues T668 and Y682-Y687 (YENPTY motif, see Fig. 5c; also present in APLP1/2), that have been reported to play a role in Aβ production^12,23,24^. Specifically, APP phosphorylated at T668 has been reported to colocalize with BACE-1 in endosomes^23^, and the YENPTY motif is known to mediate APP internalization from the plasma membrane^25^. Examining the effects of these residues in APP/BACE-1 BifC assays, we saw that the extent of APP/BACE-1 attenuation by the YENPTY mutation strongly resembled the decrease in fluorescence complementation by the APP-659 construct (Fig. 5d). A prediction from these experiments is that endocytosis of the CRISPR-mimic APP from the cell surface should be attenuated; and indeed, this was the case in internalization assays (Fig. 5e). Similar results were seen with an “APP-659-GG” construct that more closely resembles the most common post-editing translational product of our sgRNA (Supplementary Fig. 7; also see post-editing products from human cells in Fig. 2h and Supplementary Fig. 1e).

Collectively, the data suggest that our gene-editing approach does not have a major effect on post-Golgi trafficking of APP, but attenuates its endocytosis from cell surface, and consequently, its interaction with BACE-1 in endosomes—though we cannot exclude a direct effect of editing on APP/BACE-1 interaction. This is also consistent with previous studies showing that surface APP is internalized into endosomes, where it is cleaved by BACE-1^26–29^. Since most of the APP α-cleavage is thought to occur at the cell surface^30^, this may also explain why the non-amyloidogenic pathway is enhanced by our approach.

## Discussion

Using CRISPR/Cas9 technology, here we provide conceptual proof for a simple strategy that selectively edits the C-terminus of APP and alters the balance of APP-cleavage—attenuating β-cleavage and Aβ, while upregulating neuroprotective α-cleavage. The N-terminus of APP—known to play physiologic roles—is unaffected, along with the compensatory APP homologues APLP1/2. No deleterious effects were seen in neurophysiologic parameters, though our experiments were in cultured neurons and in vivo effects are unknown. Our strategy likely works by editing the terminal YENPTY motif in APP that is responsible for its internalization, subsequent APP/BACE-1 association, and initiation of the amyloidogenic pathway; while retention of APP at the plasma membrane may facilitate the upregulation of APP α-cleavage.

APP processing is regulated by α-secretases, β-secretases, and γ-secretases; and the various cleavage products may play physiological functions that are not fully understood^31,32^. Previous studies suggest that in vivo deletion of the APP C-terminus blocks APP β-cleavage without obvious effects on neuroanatomy, behavior and neuronal activity in adult mice^13^. However, long-term in vivo effects of our editing strategy are unknown, and it is not possible to definitively conclude that the physiologic function of APP is preserved in this setting. Notably however, the APP homologues APLP 1/2 also have YENPTY motifs^15,16^—that can presumably undergo endocytosis and protein–protein interactions—and are expected to compensate for the loss of the C-terminus. Though our strategy has reciprocal effects on β/α- cleavage in various cells, we have not tested the tools in an AD mouse model, which is a subject of future investigation. Also, our current off-target analyses cannot detect very small DSBs, and a more thorough analysis at a single-nucleotide resolution is warranted. The precise reasoning behind enhanced α-cleavage is also unclear. We propose that retention of APP at the plasma membrane might be responsible, but we cannot rule out other causes, including off-target effects, and further detailed studies may provide clarity.

## Methods

### Constructs, antibodies, and reagents

For transient co-expression of CRISPR/Cas9 components, *APP*-sgRNA nucleotides were synthesized and cloned into pU6-(Bbs1)_CBh-Cas9-T2A-mCherry vector at Bbs1 site. For viral transduction in neuron, a dual vector system was used to deliver CRISPR/Cas9 components using AAV9^33^. For making the AAV9 vectors, the *APP*-sgRNA was cloned into pAAV9-U6sgRNA(SapI)_hSyn-GFP-KASH-bGH vector at Sap1 site. For making the lentivirus vectors, the *APP*-sgRNA was cloned into lentiCRISPR v2 vector at Bbs1 site^34^. For making APP deletions and relevant constructs, the human APP659 truncation was PCR amplified and cloned at Hind3 and Sac2 sites of pVN to generate pAPP659:VN. The untagged APP and APP-659-GG were PCR amplified and cloned at Hind3 and Not1 sites of pcDNA3.1 vector. The pAPP^T668A^:VN and pAPP^T668A+YENPTY^:VN were generated by site directed mutagenesis from pAPP:VN and pAPP^YENPTY^:VN. Antibodies used were as follows: APP Y188 (ab32136; Abcam), APP 22C11 (MAB348; Millipore), APP 6E10 (803001; BioLegend), APP M3.2 (805701; BioLegend), APP 2E9 (MABN2295; Millipore), APP CT20 (171610; Millipore), sAPPβ (18957; IBL), BACE-1 (MAB931; R&D), GAPDH (MA5-15738, ThermoFisher), GFP (ab290, Abcam), GFP (A10262, Invitrogen), GFP (GFP-1020, Aves), HA 16B12 (901513, BioLegend), VAMP2 (104211, Synaptic Systems). Reagents were as follows: γ-secretase inhibitor BMS-299897 (Sigma), BDNF (R&D), laminin (Trevigen) and Rho Kinase (ROCK)-inhibitor H-1152P (Calbiochem).

### Cell cultures and biochemistry

HEK293 and neuro2a cells (ATCC) were maintained in DMEM with 10% FBS. Cells were transfected with Lipofectamine 2000 and collected 5 days after transfection for biochemical and immunostaining analysis. All the studies involving primary neuron culture were performed in accordance with University of Wisconsin guidelines. Primary hippocampal neurons were obtained from postnatal (P0-P1) CD1 mice (either sex), and transiently transfected using Lipofectamine 2000 or Amaxa 4D system (Lonza). Dissociated neurons were plated at a density of 30,000 cells/cm^2^ on poly-D-lysine-coated glass-bottom culture dishes (Mattek) and maintained in Neurobasal/B27 medium with 5% CO_2_. For APP/BACE-1 interaction, DIV 7 neurons were cultured for ~6–8 h after transfection. For APP transport studies, DIV 7 neurons were cultured for ~18–20 h after transfection. For spine density analysis, DIV7 neurons were transfected with Cas9, sgRNA and soluble marker, and cultured for 7 days before imaging. For testing the effect of CRISPR/Cas9 on neuronal development, neurons were electroporated with the respective constructs before plating using an Amaxa 4D-Nucleofector system with the P3 Primary Cell 4D-Nucleofector X kit S and program CL-133.

For western blotting, pre-synapse analyses and electrophysiology, DIV7 cultured neurons were infected with either AAV9-*APP*-sgRNA:GFP (2.24 × 10^13^ Vg/ml) and AAV9-Cas9 (2.4 × 10^14^ Vg/ml), or AAV9-GFP (2.58 × 10^13^ Vg/ml) and AAV9-Cas9 at a multiplicity of infection (MOI) of 1.5 × 10^5^. Neurons were analyzed 7 days post-infection. Lentivirus was produced from HEK293FT cells^35^. Briefly, HEK293FT cells (ThermoFisher) were maintained in DMEM with 10% FBS, 0.1 mM NEAA, 1 mM sodium pyruvate and 2 mM Glutamine. Cells were transfected with lentiviral-target and helper plasmids at 80–90% confluency. Two days after transfection, the supernatant was collected and filtered with 0.45 μm filter. For experiments with hESCs, H9 cells (WiCell) were cultured on a Matrigel substrate (BD Biosciences) and fed daily with TeSR-E8 culture media (StemCell Technologies). When the cells were around 60–70% confluent, they were infected with a 50/50 mixture of TeSR-E8 (with 1.0 μM H-1152P) and lentivirus supernatant. After 24 h, the virus was removed, and the cells were fed for 2 days (to recover). And then cells were treated with 0.33 μg/ml of puromycin for 72 h to select for virally-integrated hESCs. For HEK and neuro2a cell lines, cells were infected with the lentivirus carrying *APP*-sgRNA and Cas9 for 24 h. And then cells were fed for 1 day to recover. After 2 days, cells were treated with 1 μg/ml of puromycin for 72 h to select for virally-integrated cells.

Human NPCs were generated using manual rosette selection and maintained on Matrigel (Corning)^36^. Concentrated lentiviruses express control-sgRNA or hu-*APP*-sgRNA were made using Lenti-X concentrator (Clontech)^37^. The NPCs were transduced with either control-sgRNA or hu-*APP*-sgRNA after Accutase splitting and were submitted to puromycin selection the subsequent day. Polyclonal lines were expanded and treated with puromycin for 5 more days before banking. Neuronal differentiations were carried out by plating 165,000 cells/12 well-well in N2/B27 media (DMEM/F12 base) supplemented with BDNF (20 ng/ml) and laminin (1 ug/ml).

For biochemistry, cell lysates were prepared in PBS + 0.15% Triton X-100 or RIPA supplemented with protease inhibitor cocktail, pH 7.4. After centrifuging at 12,000 × *g* for 15 min at 4 °C, supernatants were quantified and resolved by SDS-PAGE for western blot analysis. For sAPPα and sAPPβ detection, cell culture medium was collected and centrifugated at 2000×*g* for 15 min at room temperature (RT). The supernatants were resolved by SDS-PAGE for western blot analysis; band intensities were measured by ImageJ. The dilution factors of antibodies for western blot were as follows: APP Y188 (1:5000), APP 22C11 (1:500), APP CT20 (1:2000), APP M3.2 (1:1000), APP 2E9 (1:2000), APP 6E10 (1:1000), sAPPβ (1:500), BACE-1 (1:500), GAPDH (1:5000), GFP ab290 (1:5000). Human Aβ40 and Aβ42 were detected using kits, according to the manufacturer’s instructions (Thermo KHB3481 and KHB3544). Briefly, supernatants from H4^single copy^ cells or human iPSC derived neurons were collected and diluted (×5 for H4 and ×2 for iPSC-neuron). The diluted supernatants and the human Aβ40/42 detection antibodies were then added into well and incubated for 3 h at RT with shaking. After washing (×4), the anti-Rabbit IgG HRP solution was added and incubated for 30 min at RT. The stabilized Chromogen was added after washing (×4) and incubated for another 30 min at RT in the dark. After addition of stop solution, absorbance at 450 nm was read using a luminescence microplate reader.

### Developing a single-copy, stable APP/BACE-1 cell line

H4 (ATCC) tetOff FlpIn empty clone was maintained in OptiMEM with 10% FBS, 200 μg/ml G418 and 300 μg/ml Zeocin. To generate an APP:VN/BACE-1:VC stable cell line carrying single copies of APP and BACE-1, the expressing plasmid and pOG44 plasmids were transfected with Lipofectamine 2000. 2 days after transfection, cells were selected with 200 μg/ml hygromycin B and 200 μg/ml G418 for 1 week. A monoclonal cell line with stable expression was selected. H4 stable cell lines were then infected with the lentivirus carrying hu-*APP*-sgRNA and Cas9, as described above. After 24 h, the virus was removed, and cells were fed for 1 day to recover. After 2 days, cells were treated with 0.7 μg/ml of puromycin for 72 h to select for virally-integrated cells.

### Generation of the APP^London^ (V717I) knockin iPSC line

CRIPSR/Cas9 was used to knockin the APP V717I mutation (APP^London^) into a commercially available control human iPSC line IMR90 (clone 4, WiCell). sgRNAs targeting Exon17 of *APP* were designed using the CRISPR design tool created by Feng Zhang’s lab and subcloned into the MLM3636 vector (AddGene). Efficacy of multiple sgRNAs was first assessed in HEK293 cells (Geneart Genomic Cleavage Detection Kit, Life Technologies). The ssDNA HDR template was designed to include a silent CRISPR blocking mutation at the PAM site of most efficacious sgRNA in addition to the APP^London^ mutation. sgRNA, Cas9-2A-mCherry (generously provided by Hynek Wichterle), and ssDNA HDR template were electroporated (Lonza nucleofector) into feeder-free IMR90 iPSCs, followed by cell sorting on mCherry signal and plating at low density on MEFs (MTI-GlobalStem). Individual clones were manually picked into a 96 well format, subsequently split into duplicate plates, one of which were used to generate sgDNA as had been done previously^38^. For each clone, exon 17 of *APP* was amplified and initially screened by restriction digest for the presence of a de novo Bcl1 site introduce by the APP^London^ mutation. Sanger sequencing was used to confirm the mutation, and successful knockin clones were expanded and banked. Potential off-target effects of CRISPR/Cas9 cleavage were analyzed by Sanger sequencing of the top 5 predicted off-target genomic locations [https://mit.crispr.edu], which demonstrated a lack of indels for multiple clones. Clone 88 was picked for future studies.

### Microscopy and image analysis

For immunostaining of endogenous APP or VAMP2, cells were fixed in 4% PFA/sucrose solution in PBS for 10 min at RT, extracted in PBS containing 0.2% Triton X-100 for 10 min at RT, blocked for 2 h at RT in 1% bovine serum albumin and 5% FBS, and then incubated with rabbit anti-APP Y188 (1:200) or mouse anti-VAMP2 (1:1000) diluted in blocking buffer for 2 h at RT. After removal of primary antibody, cells were blocked for 30 min at RT, incubated with goat anti–rabbit (Alexa Fluor 488) or goat anti–mouse (Alexa Fluor 594) secondary antibody at 1:1000 dilution for 1 h at RT and then mounted for imaging. Z-stack images (0.339 μm z-step) were acquired using an inverted epifluorescence microscope (Eclipse Ti-E) equipped with CFI S Fluor VC ×40, NA 1.30 (Nikon). An electron-multiplying charge-coupled device camera (QuantEM: 512SC; Photometrics) and LED illuminator (SPECTRA X; Lumencor) were used for all image acquisition. The system was controlled by Elements software (NIS Elements Advanced Research). Z-stacks were subjected to a maximum intensity projection. For APP Y188 staining, the average intensity of single cell body (neuro2a, HEK293 and neurons) or the whole colony (hESCs) was quantified. All the images were analyzed in Metamorph and ImageJ.

For spine density analysis^39^, DIV 7 neurons were transfected with desired constructs for 7 days, and secondary dendrites were selected for imaging. Z-stack images were captured using a ×100 objective (0.2 μm z-step) and subjected to a maximum intensity projection for analysis. For the APP/BACE-1 complementation assay, DIV 7 neurons were transfected with desired constructs for ~6–8 h and fixed. Z-stack images were captured using a ×40 objective (0.339 μm z-step) and subjected to a maximum intensity projection. The average intensity within cell bodies was quantified.

For trafficking studies in axons and dendrites, imaging parameters were set at 1 frame/s and total 200 frames. Kymographs were generated in MetaMorph, and segmental tracks were traced on the kymographs using a line tool. The resultant velocity (distance/time) and run length data were obtained for each track, frequencies of particle movements were calculated by dividing the number of individual particles moving in a given direction by the total number of analyzed particles in the kymograph, and numbers of particles per minute were calculated by dividing the number of particles moving in a given direction by the total imaging time.

For APP endocytosis assay^40^, neuro2a cells expressing APP-GFP, APP659-GFP, untagged APP or untagged APP-659-GG were starved with serum-free medium for 30 min and incubated with anti-APP (22C11, 1:100) in complete medium with 10 mM HEPES for 10 min. And then, cells were fixed, permeablized and immunostained for 22C11. The mean intensity of 22C11 along plasma membrane was calculated by dividing the total intensity along plasma membrane (=intensity of whole cell−intensity of cytoplasm) with area of plasma membrane (=area of whole cell−area of cytoplasm). The ratio of mean intensities between plasma membrane and cytoplasm was quantified.

### Stereotactic injection and histology

All the animal procedures were performed in accordance with University of Wisconsin guidelines. For in vivo injection, 1.5 μl of 1:2 AAV9 mixture of AAV9-*APP*-sgRNA:GFP (or AAV9-GFP) and AAV9-Cas9 was injected into the dentate gyrus (−2.0, ± 1.6, −1.9) of 8-week old male C57BL/6 mice (either sex)^41^. Two-weeks after surgery, the mice were sacrificed by trans-cardiac perfusion of saline, followed by 4% PFA. The brains were dissected, post-fixed with 4% PFA overnight, immersed in 30% sucrose until saturation, and sectioned at 40 μm. Sections were immunostained with following antibodies: anti-HA (1:1000, 16B12), anti-GFP (1:1000, Invitrogen) and anti-APP (1:200, Y188). Images were acquired using Zeiss LSM800 confocal microscope. Average intensities of APP staining in cell bodies was quantified using Metamorph.

### Intracerebroventricular injection and histology

All animal procedures were approved by the Mayo Institutional Animal Care and Use Committee and are in accordance with the NIH Guide for Care and Use of Laboratory animals. Free hand bilateral intracerebroventricular (ICV) injections were performed in C57BL/6 mouse pups^42^. On post-natal day 0, newborn pups were briefly cryoanesthetized on ice until no movement was observed. A 30-gauge needle attached to a 10 µl syringe (Hamilton) was used to pierce the skull of the pups just posterior to bregma and 2 mm lateral to the midline. The needle was held at a depth of approximately 2 mm, and 2 μl of a mixture of AAV9 viruses (ratio 1:2 of AAV9-*APP*-sgRNA:GFP or AAV9-GFP + AAV9-Cas9) were injected into each cerebral ventricle. After 5 min of recovery on a heat pad, the pups were returned into their home cages. Mice were sacrificed 15 days after viral injection. Animals were deeply anesthetized with sodium pentobarbital prior to transcardial perfusion with PBS, and the brain was removed and bisected along the midline. The left hemisphere was drop-fixed in 10% neutral buffered formalin (Fisher Scientific, Waltham, MA) overnight at 4 °C for histology, whereas the right hemisphere of each brain was snap-frozen and homogenized for biochemical analysis. Formalin fixed brains were embedded in paraffin wax, sectioned in a sagittal plane at 5-micron thickness, and mounted on glass slides. Tissue sections were then deparaffinized in xylene and rehydrated. Antigen retrieval was performed by steaming in distilled water for 30 min, followed by permeabilization with 0.5% Triton-X, and blocking with 5% goat serum for 1 h. Sagittal sections were then incubated with anti-GFP antibody (1:250, Aves) and anti-APP antibody (1:200, Y188) overnight at 4 °C. Sections were incubated with the secondary antibodies Alexa 488-goat anti-chicken and Alexa 568-goat anti rabbit (1:500, Invitrogen) for 2 h at RT. Sections were washed and briefly dipped into 0.3% Sudan Black in 70% ethanol prior to mounting.

### Electrophysiology

A coverslip with cultured cells at a density of 60,000 cells/cm^2^ was placed in a continuously perfused bath, viewed under IR-DIC optics and whole-cell voltage clamp recordings were performed (−70 mV, room temp.). The extracellular solution consisted of (in mM): 145 NaCl, 2.5 KCl, 1 MgCl_2_, 2 CaCl_2_, 10 HEPES, and 10 dextrose, adjusted to 7.3 pH with NaOH and 320 mOsm with sucrose. Whole-cell recordings were made with pipette solutions consisting of (in mM) 140 KCl, 10 EGTA, 10 HEPES, 2 Mg2-ATP, and 20 phosphocreatine, adjusted to pH 7.3 with KOH and 315 mOsm with sucrose. Excitatory synaptic events were isolated by adding 10 µM bicuculline to block GABA (subscript A) receptors. Miniature synaptic events were isolated by adding 100 nM tetrodotoxin to prevent action potentials. mEPSCs were detected using the template-matching algorithm in Axograph X, with a template that had 0.5 ms rise time and 5 ms decay. Statistics were computed using the Statistics Toolbox of Matlab.

### T7 endonuclease I assay, off-target, and ICE analyses

Genomic PCR was performed around each sgRNA target, and related off-target sites, following the manufacturer’s instructions (using AccuPrime HiFi Taq with 500 ng of genomic DNA). Products were then purified using Wizard SV Gel and PCR Clear-Up System (Promega), and quantified using a Qubit 2.0 (Thermo Fischer). T7EI assay was performed according to manufacturer’s instructions (New England Biolabs). Briefly, 200 ng of genomic PCR was combined with 2 μl of NEBuffer 2 (New England Biolabs) and diluted to 19 μl. Products were then hybridized by denaturing at 95 °C for 5 min then ramped down to 85 °C at −2 °C/s. This was followed by a second decrease to 25 °C at −0.1 °C/s. To hybridized product, 1 μl T7EI (M0302, New England Biolabs) was added and mixed well followed by incubation at 37 °C for 15 min. Reaction was stopped by adding 1.5 μl of 0.25 M EDTA. Products were analyzed on a 3% agarose gel and quantified using a Gel Doc XR system (BioRad). Off-target sites were identified and scored using Benchling [www.benchling.com]. The top 5 off-target sites—chosen on the basis of raw score and irrespective of being in a coding region—were identified and analyzed using T7EI assay. For TIDE^43^, PCR was performed on genomic DNA using Accuprime Taq HiFi (Thermo Fischer) according to manufacture specifications. Briefly, reactions were cycled at 2 min at 94 °C followed by 35 cycles of 98 °C for 30 s, 58 °C for 30 s, and 68 °C for 2 min 30 s and a final extension phase of 68 °C for 10 min. Products were then subjected to Sanger Sequencing and analyzed using the TIDE platform [https://tide.nki.nl/]. The primers used for TIDE analyses are listed in Supplementary Table 2. For analyses of indel after CRISPR editing with *APP*670-sgRNA and *APP*676-sgRNA, the edited regions of genomic DNA were PCR amplified and subjected to Sanger Sequencing. The results were analyzed using the ICE platform [https://www.synthego.com/products/bioinformatics/crispr-analysis].

### Deep sequencing sample preparation and data analysis

Genomic PCR was performed using AccuPrime HiFi Taq (Life Technologies) following manufacturer’s instructions. About 200–500 ng of genomic DNA was used for each PCR reaction. Products were then purified using AMPure XP magnetic bead purification kit (Beckman Coulter) and quantified using a Nanodrop2000. Individual samples were pooled and run on an Illumina HiSeq2500 High Throughput at a run length of 2 × 125 bp. A custom python script was developed to perform sequence analysis. For each sample, sequences with frequency of less than 100 reads were filtered from the data. Sequences in which the reads matched with primer and reverse complement subsequences classified as target sequences. These sequences were then aligned with corresponding wildtype sequence using global pairwise sequence alignment. Sequences that were misaligned through gaps or insertions around the expected cut site were classified as NHEJ events. The frequency, length, and position of matches, insertions, deletions, and mismatches were all tracked in the resulting aligned sequences.

### Statistical analysis

Statistical analysis was performed and plotted using Prism software. Student’s *t*-test (unpaired, two-tailed) was used to compare two groups. One-way ANOVA test was used to compare multiple groups, following with Tukey multiple comparison test of every pair. A *P*-value < 0.05 was considered significant.

### Reporting summary

Further information on experimental design is available in the Nature Research Reporting Summary linked to this article.

## Supplementary information

Supplementary Information

Reporting Summary

Source Data

## Data Availability

Raw reads from sequencing is available at NCBI Bioproject PRJNA417829. The authors declare that all other data supporting the findings of this study are available within the article and its Supplementary Information files or are available from the authors upon request. The source data underlying Figs. 1b-e, 2c-e, 3b, 3d-f, 4d, 4g, 5a-b, 5d-e and Supplementary Figs. 1b-d, 2a-c, 3a-g, 4c, 5c-e, 6b-c and 7b-c are provided as a Source Data file.
