# Supplementary material for: CRISPR/Cas9 editing of APP C-terminus attenuates β-cleavage and promotes α-cleavage
Source: Nat Commun. 2019 Jan 3;10:53. doi: 10.1038/s41467-018-07971-8 (PMC6318289; doi:10.1038/s41467-018-07971-8)
Supplement: Supplementary file 1 — Supplementary Information [file 41467_2018_7971_MOESM1_ESM.pdf]

Supplementary Information

CRISPR/Cas9 editing of APP C-terminus attenuates β-cleavage and promotes α-cleavage

Sun et al.

Supplementary Fig. 1

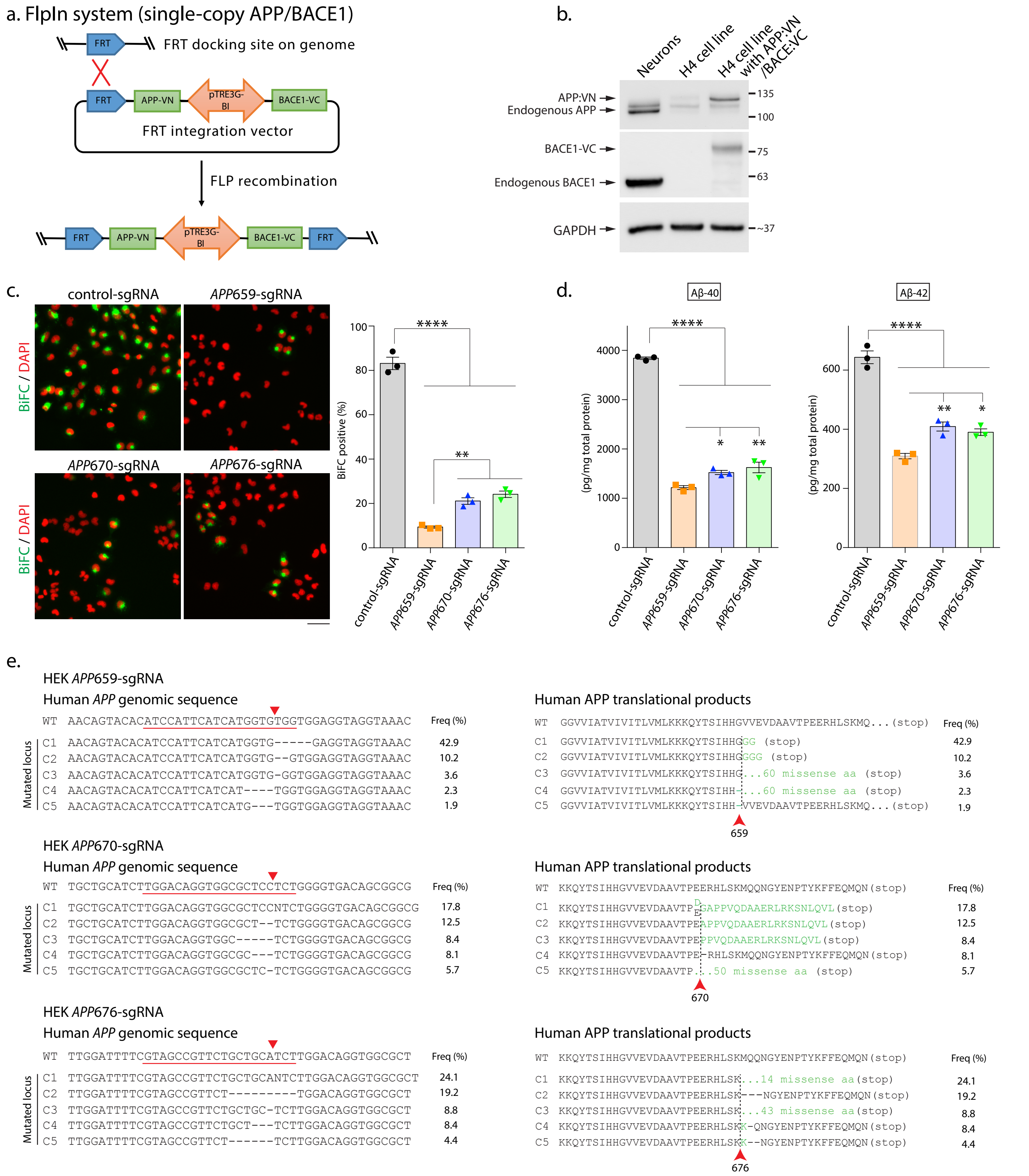

Supplementary Fig. 1. The choice of CRISPR editing site at APP C-terminus.

(a) Strategy to integrate APP:VN and BACE-1:VC into the H4 genome and generation of a stable cell line expressing single copies of the two proteins (see results and methods for details).

(b) APP and BACE-1 expression in the H4$^{\text{single copy}}$ cell line. Note negligible expression of endogenous proteins in native H4 cells.

(c) The H4$^{\text{single copy}}$ cell line was transduced with lentiviral vectors carrying non-targeting control-sgRNA/Cas9 or various human APP C-terminus targeting sgRNAs/Cas9 (see **Supplementary Table 1** for targeting sequences). The

APP/BACE-1 Venus complementation was visualized by fluorescence microscopy. Note attenuation of complemen--tation, indicating editing by the *APP*-sgRNAs (quantified on right, mean ± SEM of three independent experiments). One-way ANOVA: p<0.0001. Tukey's multiple comparisons: p<0.0001 (control-sgRNA vs *APP*659-sgRNA); p<0.0001 (control-sgRNA vs *APP*670-sgRNA); p<0.0001 (control-sgRNA vs *APP*676-sgRNA); p=0.0064 (*APP*659-sgRNA vs *APP*670-sgRNA); p=0.0015 (*APP*659-sgRNA vs *APP*676-sgRNA); p=0.6207 (*APP*670-sgRNA vs *APP*676-sgRNA). Scale bar 50 µm.

(d) ELISA of media from the H4single copy cell line (treated as above). Note decreased Aβ in the *APP*-sgRNAs treated samples (mean ± SEM of three independent experiments). One-way ANOVA for Aβ 40 and 42: p<0.0001. Tukey's multiple comparisons for Aβ 40: p<0.0001 (control-sgRNA vs *APP*659-sgRNA; control-sgRNA vs *APP*670-sgRNA; control-sgRNA vs *APP*676-sgRNA); p=0.0331 (*APP*659-sgRNA vs *APP*670-sgRNA); p=0.0071 (*APP*659-sgRNA vs *APP*676-sgRNA); p=0.6673 (*APP*670-sgRNA vs *APP*676-sgRNA). Tukey's multiple comparisons for Aβ 42: p<0.0001 (control-sgRNA vs *APP*659-sgRNA; control-sgRNA vs *APP*670-sgRNA; control-sgRNA vs *APP*676-sgRNA); p=0.0068 (*APP*659-sgRNA vs *APP*670-sgRNA); p=0.0221 (*APP*659-sgRNA vs *APP*676-sgRNA); p=0.8079 (*APP*670-sgRNA vs *APP*676-sgRNA).

(e) HEK cells were transduced by lentiviral vectors carrying *APP*-sgRNAs and Cas9 (or non-targeting control-sgRNA/Cas9 as control), and APP C-terminus was sequenced. Left: Deep sequencing of *APP*659-sgRNA treated cells, and Sanger sequencing followed by ICE analyses for *APP*670-sgRNA and *APP*676-sgRNA treated cells. Red underlines mark the sgRNA-targeting sequences and arrowheads denote predicted cut-sites. Right: Predicted APP translational products after CRISPR/Cas9 editing in human HEK cells for the major mutant alleles observed in sequencing analyses. Red arrowheads indicate the amino acids where *APP* genes were translated up to after editing).

Source data are provided as a Source Data file.

Supplementary Fig. 2

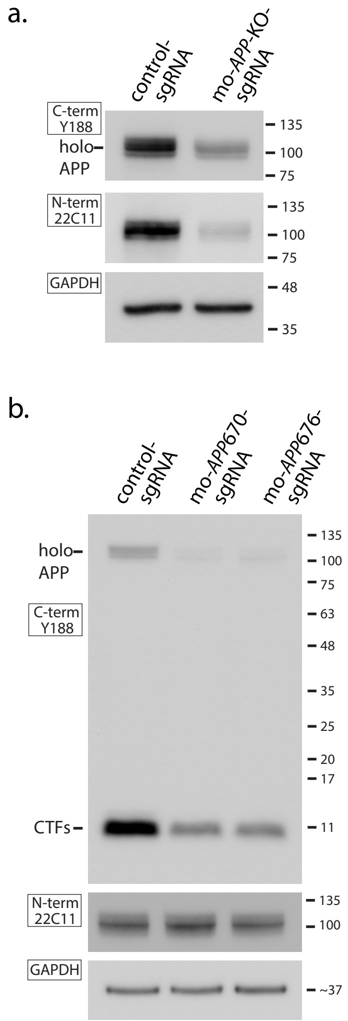

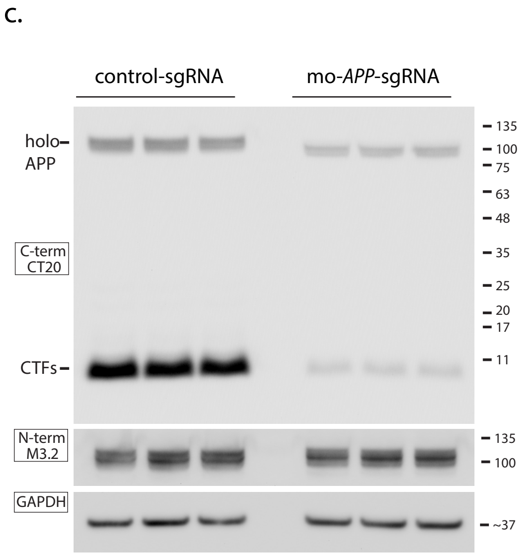

d. Mouse translational products (post-editing)

Mouse APP translation (neuro2a)

			Freq (%)
WT	GGVVIATVIVITLVMLKKKQYTSIHHGVVEVDAAVTPEERHLSKMQ...	(stop)	
C1	GGVVIATVIVITLVMLKKKQYTSIHHGGGRRRRRDPRGAPSLQDAAERI	(stop)	22.4
C2	GGVVIATVIVITLVMLKKKQYTSIHHGGGRRRRRDPRGAPSLQDAAERI	(stop)	6.8
C3	GGVVIATVIVITLVMLKKKQYTSIHHGGRRRRDPRGAPSLQDAAERI	(stop)	5.9
C4	GGVVIATVIVITLVMLKKKQYTSIHHGGGGRRRRRDPRGAPSLQDAAERI	(stop)	3.8
C5	GGVVIATVIVITLVMLKKKQYTSIHHGGGGRRRRRDPRGAPSLQDAAERI	(stop)	3.4

659

Supplementary Fig. 2. Evaluation of CRISPR editing by immunoblotting in mouse neuro2a cells.

(a) Neuro2a cells were co-transfected with a sgRNA that knocked out the entire *APP* gene and Cas9 (see **Supplementary Table 1** for targeting sequence), and immunostained with APP N-terminal and C-terminal antibodies (after 5 days in culture). Note attenuation of staining for both Y188 and 22C11.

(b) Neuro2a cells were transfected with non-targeting control-sgRNA or various APP C-terminus targeting sgRNAs (see **Supplementary Table 1** for targeting sequences), and immunostained with APP N-terminal and C-terminal antibodies (after 5 days in culture in the presence of GSI). Note attenuation of staining by Y188 but not 22C11, indicating selective editing of the APP C-terminus.

(c) Neuro2a cells were transduced by lentiviral vectors carrying *APP*-sgRNA and Cas9 (or non-targeting control-sgRNA/Cas9 as control) and immunoblotted with the APP antibodies CT20 and M3.2 (CT20 recognizes last 20 aa; M3.2 recognizes an extracellular domain located upstream of the CRISPR/Cas9 targeting site). A GSI was added to allow detection of accumulated APP CTF's. Note attenuated signal with CT20- but not M3.2- antibody, indicating selective editing of the APP C-terminus.

(d) Post-editing translational products in mouse (neuro 2a) cells. Note effective truncation of APP at aa 659.

Source data are provided as a Source Data file.

Supplementary Fig. 3

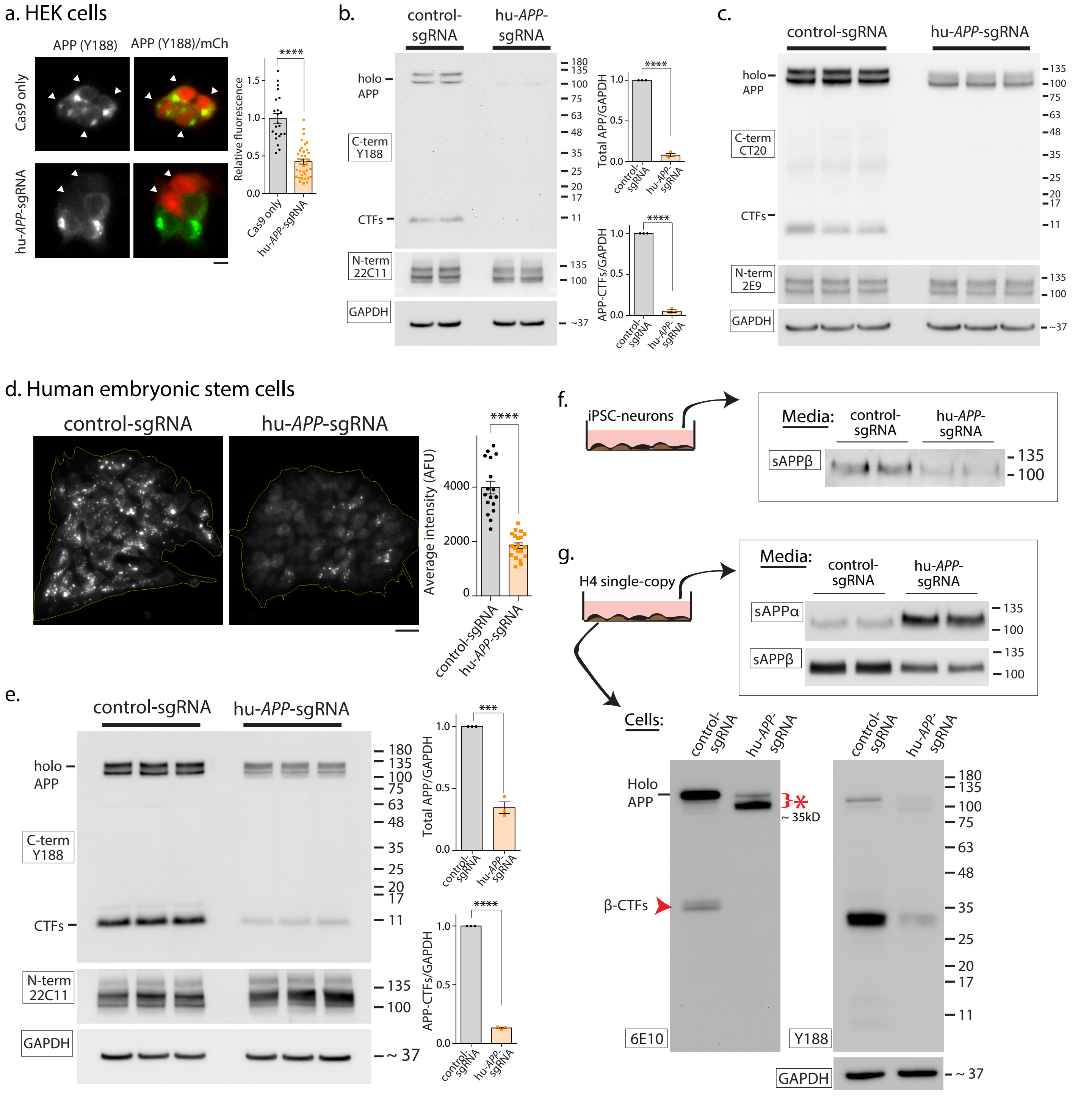

Supplementary Fig. 3. APP C-terminus editing by CRISPR/Cas9.

(a) HEK cells were transfected with human-specific *APP*-sgRNA and Cas9 (or Cas9 only), and immunostained with the Y188 antibody (after 5 days in culture). Note attenuation of staining, quantified on right (mean ± SEM of 25 cells for Cas9 only and 43 cells for hu-*APP*-sgRNA from two independent experiments). Scale bar 10 µm.

(b) HEK cells were transduced by lentiviral vectors carrying *APP*-sgRNA and Cas9 (or non-targeting control-sgRNA/Cas9 as control) and immunoblotted with the Y188 and 22C11 antibodies (in the presence of GSI). Note attenuation of APP-CTFs in *APP*-sgRNA treated cells, indicating CRISPR-editing (mean ± SEM of three independent experiments).

(c) HEK cells above were immunoblotted with CT20 and 2E9 antibodies (CT20 recognizes last 20 aa; 2E9 recognizes APP extracellular domain upstream of the CRISPR/Cas9 targeting site). Note attenuated signal with CT20- but not 2E9- antibody, indicating selective editing of the APP C-terminus.

(d, e) Human ESCs were transduced by lentiviral vectors carrying human *APP*-sgRNA/Cas9 (or non-targeting sgRNA/Cas9). Samples were immunostained with the Y188 antibody (d) or immunoblotted with the Y188 and 22C11 antibodies (e). Note attenuation of

APP-CTFs in sgRNA-transduced group (for immunostaining, mean ± SEM of 17 colonies for control-sgRNA and 20 colonies for hu-*APP*-sgRNA from two independent experiments; for western blotting, mean ± SEM of three independent experiments). Scale bar 20 µm.

(f) Media from iPSC derived neurons were immunoblotted for extracellular sAPPβ (in the absence of GSI). Note decrease in APP β-cleavage in the hu-*APP*-sgRNA treated samples.

(g) Media from H4$^{single\text{-}copy}$ cells were immunoblotted for extracellular sAPPα with 6E10 antibody and sAPPβ (in the absence of GSI). Note enhanced APP α-cleavage and attenuated APP β-cleavage in the hu-*APP*-sgRNA treated samples.

For all panels, significance determined with two-tailed t-test, *** $p<0.001$, **** $p<0.0001$. Source data are provided as a Source Data file.

Supplementary Fig. 4

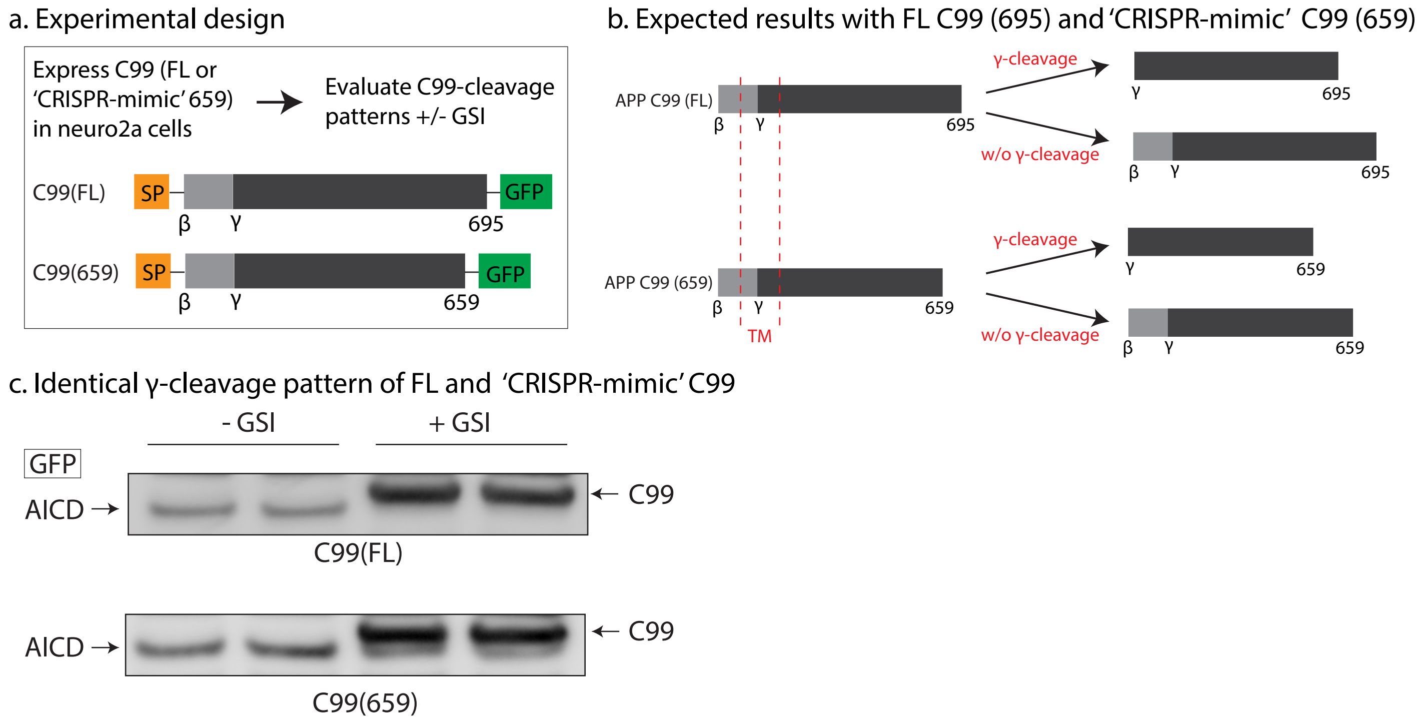

Supplementary Fig. 4. Gene editing by *APP*-sgRNA likely does not influence APP γ-cleavage.
(a) Strategy to evaluate γ-cleavage of post-edited APP. Neuro2a cells were transfected with either full length (FL) C99, or C99 truncated at aa 659 (to mimic the post-editing translational product; all constructs were GFP-tagged to confirm expression). γ-cleavage of the FL and 659 C99 was evaluated by western blotting (note that neuro2a cells have all components of the γ-secretase complex).
(b) Schematic showing expected C99-cleavage patterns. Note that upon γ-cleavage, both C99-fragments will be further truncated. However, if the 'CRISPR-mimic' (659) fragment did not undergo γ-cleavage, this truncation would not occur.
(c) Western blotting of the cells from (a) indicates that both C99 fragments (FL and 659) undergo γ-cleavage – as indicated by the shift upon inhibiting γ-cleavage by GSI. These data suggest that gene editing by the *APP*-sgRNA likely does not affect APP γ-cleavage, and that the effects seen on the amyloid pathway are likely due to modulation of APP-β-cleavage. Source data are provided as a Source Data file.

Supplementary Fig. 5

a. Off-target sites

		Sequence	Location	Coding region
Mouse	OT1	GTCCATCCATCATGGCCTGG	chr18:+82500985	No
	OT2	TTCCATCCATCATGGCTTGG	chr15:+83079515	No
	OT3	GTCCCTCCATCATGGCCTGG	chr8:+54570804	No
	OT4	GGCCATCATTCATGGCGTGG	chr12:-30225859	No
	OT5	ATAAATATATCATGGCGTGG	chr2:+85075854	No
Human	OT1	CTCCCTTCATCTTGGTGTGG	chr17:+42059975	No
	OT2	ATGCAATCAGCATGGTGTGG	chr11:+45935195	Yes, *PHF21A*
	OT3	TTCTGTTCAGCATGGTGTGG	chr19:+13830088	No
	OT4	TTAAATTCAACATGGTGTGG	chrX:+78686020	No
	OT5	AGCCATTTATCAAGGTGTGG	chr8:+61517706	No

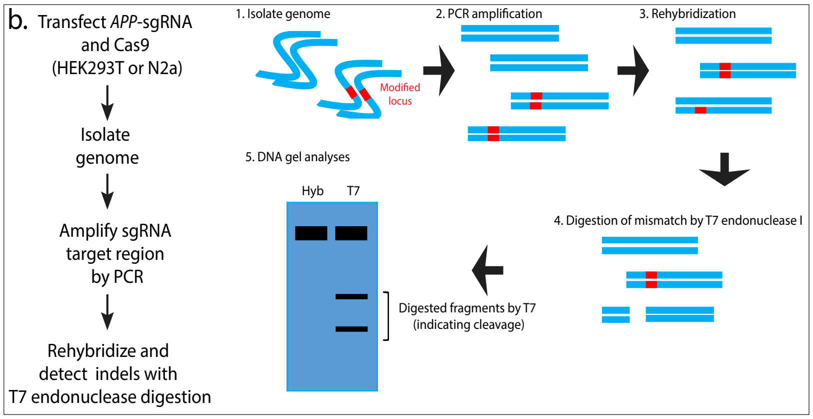

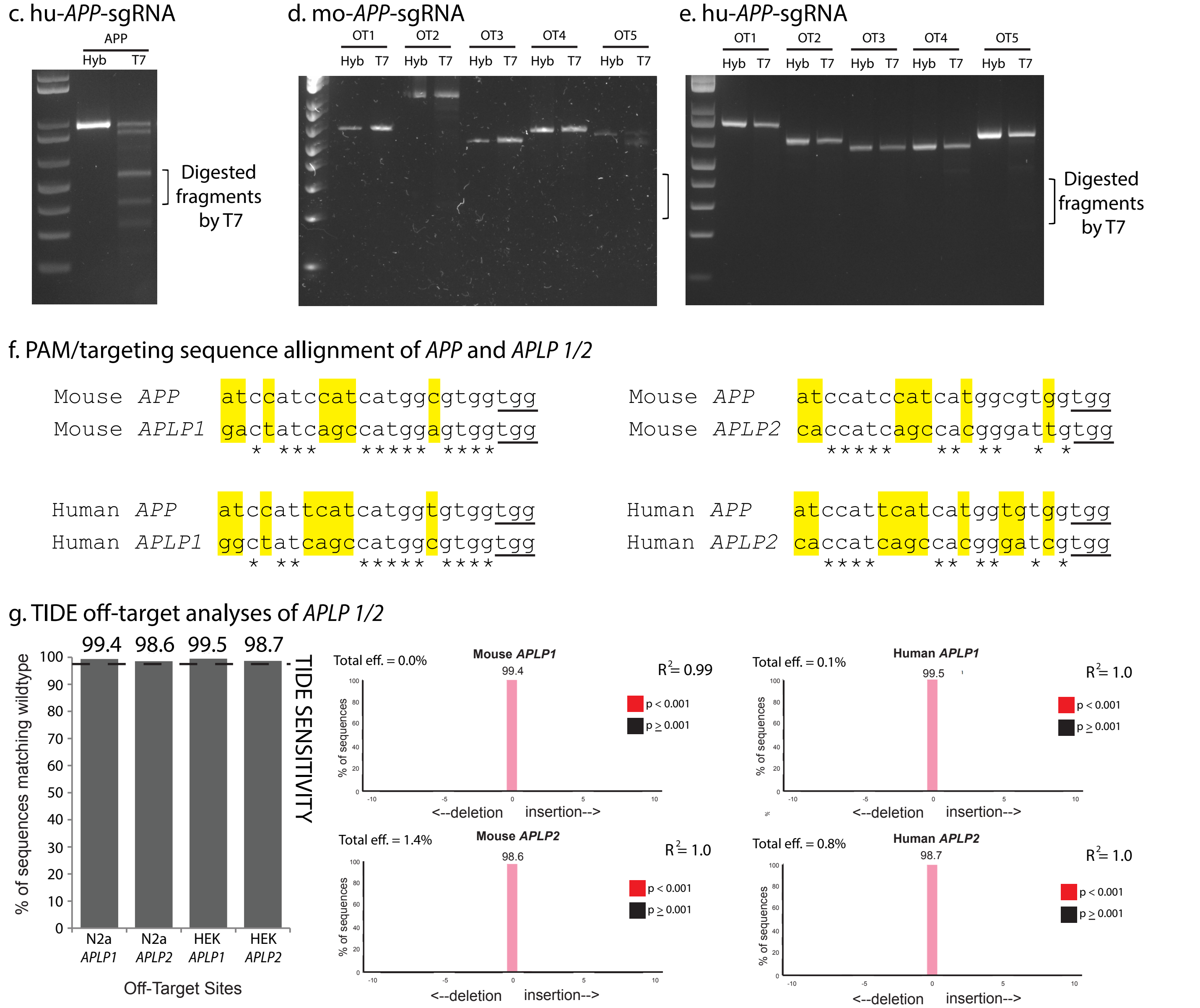

Supplementary Fig. 5. Off target analyses of *APP*-sgRNA.

(a) Computationally predicted top five off-target (OT) sites in the genome, that can be potentially targeted by the mouse and human *APP*-sgRNAs (mismatched nucleotides in the targeting sequence are marked in red). Genomic locations corresponding to the sequences is shown on the right column (note most are in non-coding regions).

(b) Strategy of T7 endonuclease digestion assay to detect genome-editing events. Genomic DNA was PCR amplified with primers bracketing the modified locus. PCR products were then rehybridized, yielding three possible structures. Duplexes

containing a mismatch were digested by T7 endonuclease I. DNA gel analysis was used to calculate targeting efficiency. Note digested fragments in the gel indicates cleavage.

(c) Gene edits at the *APP* locus by the *APP*-sgRNA, as seen by T7 endonuclease digestion. Note two digested fragments were recognized after T7 endonuclease digestion.

(d, e) T7 endonuclease assays of potential off-target sites (mouse and human). No digested fragments are seen, indicating that the sgRNAs do not generate detectable gene edits at these sites.

(f) Comparison of *APLP1* and *APLP2* sequences with *APP* at the sgRNA targeting site. Asterisks mark conserved nucleotide sequences, and the PAM sites are underlined. Nucleotide mis-matches are highlighted in yellow. Note extensive mis-match of the mouse and human sequences at the sgRNA targeting site.

(g) Left: Off-target TIDE analysis of APP family members *APLP1* and *APLP2* in mouse (neuro2a) and human (HEK) cell lines following lentiviral integration of Cas9 using TIDE. No modifications were detected below the TIDE limit of detection (dotted line) in either of the populations, indicating that the *APP*-sgRNA was unable to edit *APLP 1/2*. Right: TIDE analysis of *APLP1* and *APLP2* loci in mouse and human cell lines. Neither of the populations had significant editing at either of the two loci, and all sequences had a near perfect correlation to the model.

Source data are provided as a Source Data file.

Supplementary Fig. 6

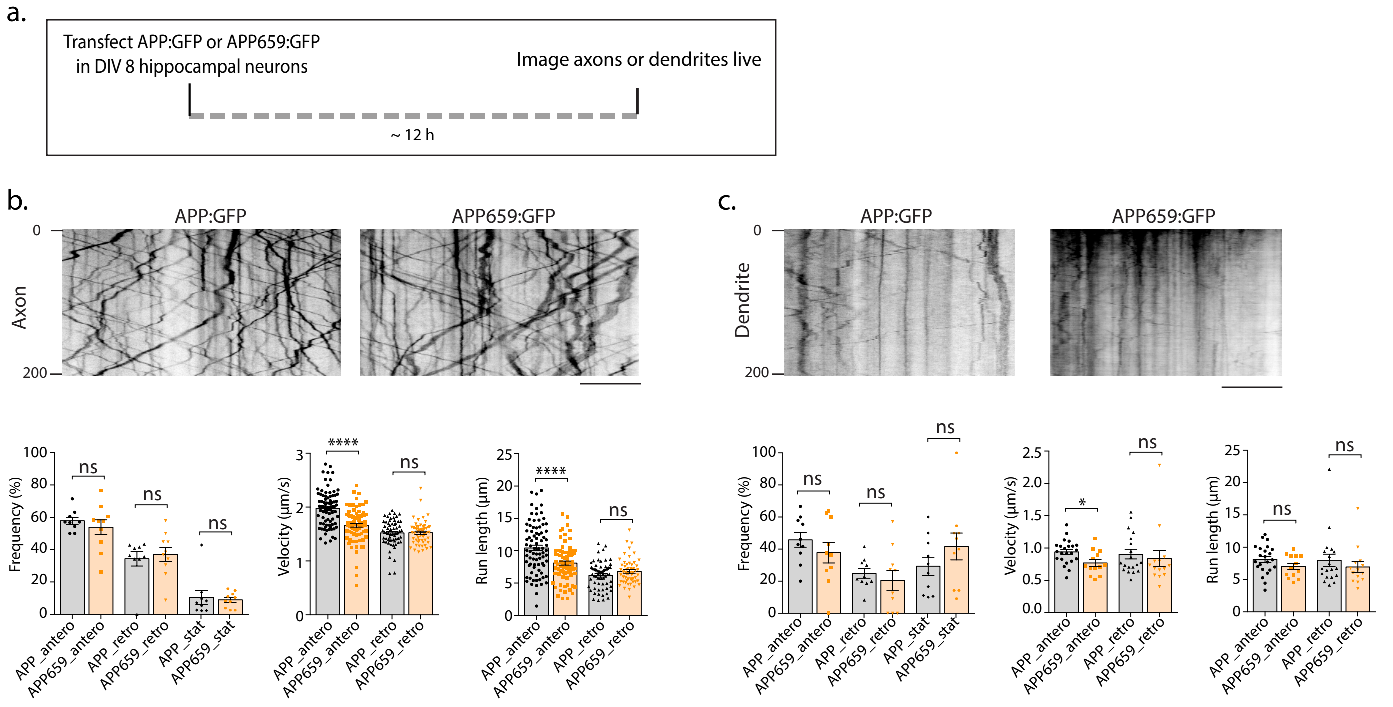

Supplementary Fig. 6. Trafficking of vesicles carrying APP(WT) or APP(659).
(a) Cultured hippocampal neurons were transfected with APP(WT):GFP or APP(659):GFP, and kinetics of APP particles were imaged live in axons and dendrites.
(b) Representative kymographs and quantification of APP kinetics in axons. Note that there was no change in frequency of transport, and only a modest reduction in run-length and velocity. Error bars, mean ± SEM of 325 APP(WT):GFP and 310 APP(659):GFP vesicles in 10-12 neurons from two independent experiments. Significance determined with two-tailed t-test. Frequency: p=0.4635 (APP_antero vs APP659_antero); p=0.6650 (APP_retro vs APP659_retro); p=0.7420 (APP_stat vs APP659_stat). Velocity: P<0.0001 (APP_antero vs APP659_antero); p=0.9419 (APP_retro vs APP659_retro). Run length: p<0.0001 (APP_antero vs APP659_antero); p=0.2433 (APP_retro vs APP659_retro). Scale bar 5 µm.
(c) Representative kymographs and quantification of APP kinetics in dendrites. Error bars, mean ± SEM of 130 APP(WT):GFP and 115 APP(659):GFP particles in 10-12 neurons from two independent experiments. Significance determined with two-tailed t-test. Frequency: p=0.3245 (APP_antero vs APP659_antero); p=0.5438 (APP_retro vs APP659_retro); p=0.2394 (APP_stat vs APP659_stat). Velocity: p=0.0120 (APP_antero vs APP659_antero); p=0.6248 (APP_retro vs APP659_retro). Run length: p=0.1352 (APP_antero vs APP659_antero); p=0.4284 (APP_retro vs APP659_retro). Scale bar 5 µm.
Source data are provided as a Source Data file.

Supplementary Fig. 7

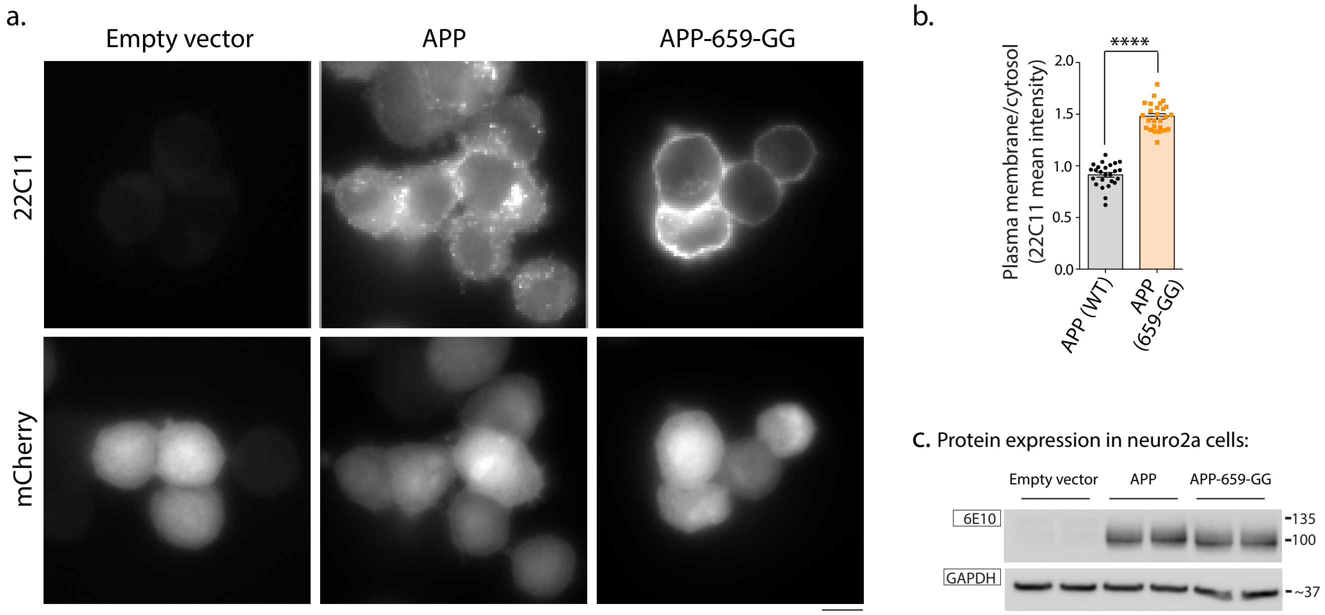

Supplementary Fig. 7. Internalization of APP-659-GG (most common post-editing translational product).
(a,b) Neuro2a cells were co-transfected with untagged APP-659-GG and mCherry (or untagged WT APP and mCherry as control). After incubation with anti N-terminal APP antibody (22C11) for 10 min, the cells were fixed and stained with secondary antibody to visualize surface and internalized APP (mCherry labels transfected cells). Note accumulation of APP-659-GG on the cell surface, along with decreased internalization; quantified in (b). Mean ± SEM of 25 cells for APP(WT) and 26 cells for APP-659-GG from two independent experiments, $p<0.0001$ by two-tailed t-test. Scale bar 10 µm.
(c) Expression levels of exogeneous APP constructs. Note that WT and APP-659-GG were expressed at similar levels in the neuro2a cells above.
Source data are provided as a Source Data file.

Supplementary Table 1

APP-sgRNAs targeting sequences

	sgRNA targeting sequence
Human *APP* 659	ATCCATTCATCATGGTGTGG
Human *APP* 670	TGGACAGGTGGCGCTCCTCT
Human *APP* 676	GTAGCCGTTCTGCTGCATCT
Mouse *APP* 659	ATCCATCCATCATGGCGTGG
Mouse *APP* 670	TGGAGAGATGGCGCTCCTCT
Mouse *APP* 676	ATATCCGTTCTGCTGCATCT
Mouse *APP* KO	CGCCTGGACGGTTCGGGCTC

Supplementary Table 2

PCR primers used for on- and off-target genomic loci amlification

	Forward primer sequence (5'-3')	Reverse primer sequence (5'-3')
Mouse *APP* (659)	AGGAACGGAGTGACCTGTTTCC	TTCCTCCATGGTAACCACGCAT
Human *APP* (659)	TGGGGAAGCCACATGTTGTACA	ATGTTTTGGTGGGCCATTTGGT
Human *APP* (670; 676)	AAATTATGGGTGTTCTGCAATCTTGG	ACTTGTGTTACAGCACAGCTGTC
Mouse OT1	GCCCTCCAGAAGTATTGGCTT	GTCAGGGCCTTGCTCTACAAA
Mouse OT2	CGCAAAAACTGGCTGCGTAT	TGTAGGCGCACATGCAGAAG
Mouse OT3	CAGGTAGAGCGTGGAAACTCA	TGTGCGCATTAGGACCAGAT
Mouse OT4	CACCTGACAATGCTGTCCCA	AGACAAGGTCTGTCTCCTTGC
Mouse OT5	CCAACTCTTTGCTTAGGGGC	ATCGTCCCTGGTGCATTCTC
Human OT1	GGAAAACCAGGTAGAGGGGG	TCTCTGGCTCGAGGGTACAT
Human OT2	CTGCATGCCATGGGTAGGTA	CAGGCTGTTTCGGGTCCTT
Human OT3	AGACTCTTCTCCGATTCCAGC	TCCAGCACGATCTGGTAGGC
Human OT4	AGTGCTTTTCTTTGCCTTTGCT	TGCTCGGGAGGTGTTTCTAC
Human OT5	AACAAGGCAGCTCCTCAACT	GACGTCAGAATTGAGGGTGGA
Mouse *APLP1*	CCAGCGGGATGAACTGGTAAGA	CCCAGGTCACCTTAAGGAGCAA
Mouse *APLP2*	GAGAGAGTTGGAGGCCTTGAGG	AACCACAGTGACAAGTGGCTCT
Human *APLP1*	GTGAATGCGTCTGTTCCAAGGG	GCTGCTGGGACTATCTGGGAAT
Human *APLP2*	TTTTAGGGGCTCGACCTTCCAG	TGCACTAATTTCCCAGGGCTCA

Supplementary Table 3

Transport parameters of WT and APP659

Kinetics in axons								
	% Anterograde	% Retrograde	% Stationary	Anterograde Run-length (µm)	Retrograde Run-length (µm)	Anterograde velocity (µm/sec), mean±SEM	Reterograde velocity (µm/sec), mean±SEM	
APP659	53.88 ± 4.57	37.13 ± 4.36	8.97 ± 1.51	8.08 ± 0.31	6.8 ± 0.26	1.66 ± 0.03	1.52 ± 0.03	
APPWT	57.84 ± 2.22	34.37 ± 4.46	10.42 ± 4.24	10.44 ± 0.42	6.35 ± 0.26	1.97 ± 0.03	1.52 ± 0.03	
Kinetics in dendrites								
	% Anterograde	% Retrograde	% Stationary	Anterograde Run-length (µm)	Retrograde Run-length (µm)	Anterograde velocity (µm/sec), mean±SEM	Reterograde velocity (µm/sec), mean±SEM	
APP659	37.81 ± 6.45	20.55 ± 6.21	41.64 ± 8.37	7.0 ± 0.48	6.94 ± 0.81	0.76 ± 0.05	0.83 ± 0.12	
APPWT	45.83 ± 4.58	24.81 ± 2.97	29.35 ± 5.63	8.12 ± 0.46	7.98 ± 0.94	0.94 ± 0.03	0.9 ± 0.07	
~ 115 APP659:GFP and ~ 130 APP:GFP vesicles analyzed in dendrites; ~ 310 APP659:GFP and ~325 APP:GFP vesicles in axons (from 10-12 neurons from 2 separate cultures.)								
