# Supplementary material for: CRISPR/Cas9 editing of APP C-terminus attenuates β-cleavage and promotes α-cleavage
Source: Nat Commun. 2019 Jan 3;10:53. doi: 10.1038/s41467-018-07971-8 (PMC6318289; doi:10.1038/s41467-018-07971-8)
Supplement: Supplementary file 2 — Reporting Summary [file 41467_2018_7971_MOESM2_ESM.pdf]

nature research

Corresponding author(s): Subhojit Roy

Reporting Summary

Nature Research wishes to improve the reproducibility of the work that we publish. This form provides structure for consistency and transparency in reporting. For further information on Nature Research policies, see Authors & Referees and the Editorial Policy Checklist.

Statistical parameters

When statistical analyses are reported, confirm that the following items are present in the relevant location (e.g. figure legend, table legend, main text, or Methods section).

n/a	Confirmed	
☐	☒	The exact sample size (n) for each experimental group/condition, given as a discrete number and unit of measurement
☐	☒	An indication of whether measurements were taken from distinct samples or whether the same sample was measured repeatedly
☐	☒	The statistical test(s) used AND whether they are one- or two-sided<br>*Only common tests should be described solely by name; describe more complex techniques in the Methods section.*
☐	☒	A description of all covariates tested
☐	☒	A description of any assumptions or corrections, such as tests of normality and adjustment for multiple comparisons
☐	☒	A full description of the statistics including central tendency (e.g. means) or other basic estimates (e.g. regression coefficient) AND variation (e.g. standard deviation) or associated estimates of uncertainty (e.g. confidence intervals)
☐	☒	For null hypothesis testing, the test statistic (e.g. F, t, r) with confidence intervals, effect sizes, degrees of freedom and P value noted<br>*Give P values as exact values whenever suitable.*
☒	☐	For Bayesian analysis, information on the choice of priors and Markov chain Monte Carlo settings
☒	☐	For hierarchical and complex designs, identification of the appropriate level for tests and full reporting of outcomes
☒	☐	Estimates of effect sizes (e.g. Cohen's d, Pearson's r), indicating how they were calculated
☐	☒	Clearly defined error bars<br>*State explicitly what error bars represent (e.g. SD, SE, CI)*

Our web collection on statistics for biologists may be useful.

Software and code

Policy information about availability of computer code

Data collection	Microscopy imaging softwares: MetaMorph 1.10.1.161, NIS AR4.20.02, and Zen 2.3. Western blot imaging software: Vision Works 8.19.17027.9424. Next generation sequencing data was collected with built-in software with Illumina HiSeq2500.
Data analysis	Image J, MetaMorph, and GraghPad Prism.

For manuscripts utilizing custom algorithms or software that are central to the research but not yet described in published literature, software must be made available to editors/reviewers upon request. We strongly encourage code deposition in a community repository (e.g. GitHub). See the Nature Research guidelines for submitting code & software for further information.

Data

Policy information about availability of data

All manuscripts must include a data availability statement. This statement should provide the following information, where applicable:

- Accession codes, unique identifiers, or web links for publicly available datasets
- A list of figures that have associated raw data
- A description of any restrictions on data availability

Raw reads from sequencing is available at NCBI Bioproject PRJNA417829. The authors declare that all other data supporting the findings of this study are available

within the article and its Supplementary Information files or are available from the authors upon request. The source data underlying Figs 1b-e, 2c-e, 3b, 3d-f, 4d, 4g, 5a-b, 5d-e and Supplementary Figs 1b-d, 2a-c, 3a-g, 4c, 5c-e, 6b-c and 7b-c are provided as a Source Data file.

Field-specific reporting

Please select the best fit for your research. If you are not sure, read the appropriate sections before making your selection.

☒ Life sciences ☐ Behavioural & social sciences ☐ Ecological, evolutionary & environmental sciences

For a reference copy of the document with all sections, see nature.com/authors/policies/ReportingSummary-flat.pdf

Life sciences study design

All studies must disclose on these points even when the disclosure is negative.

Sample size	Sample size was calculated based on similar studies in the literature.
Data exclusions	We did not exclude any data points.
Replication	Experiments were performed using sufficient number of biological replicates to ensure reproducibility. All the results were successfully reproduced.
Randomization	The experiment was not randomized. Each experiment was performed with appropriate control.
Blinding	Blinding was done for the analysis of neuron physiology (Figure 3g) and APP staining with hippocampi injection of APP CRISPR viruses (Figure 3c and 3d). No blinding was done for other experiments and analysis as they were performed using unbiased methods.

Reporting for specific materials, systems and methods

Materials & experimental systems

n/a	Involved in the study	
☒	☐	Unique biological materials
☐	☒	Antibodies
☐	☒	Eukaryotic cell lines
☒	☐	Palaeontology
☐	☒	Animals and other organisms
☒	☐	Human research participants

Methods

n/a	Involved in the study	
☒	☐	ChIP-seq
☒	☐	Flow cytometry
☒	☐	MRI-based neuroimaging

Antibodies

Antibodies used	APP Y188 (ab32136; Abcam), APP 22C11 (MAB348; Millipore), APP 6E10 (803001; BioLegend), APP M3.2 (805701; BioLegend), APP 2E9 (MABN2295; Millipore), APP CT20 (171610; Millipore), sAPP-beta(18957; IBL) BACE-1 (MAB931; R&D), GAPDH (MA5-15738, ThermoFisher), GFP (ab290, Abcam), GFP (A10262, Invitrogen), HA (901513, BioLegend), VAMP2 (104211, Synaptic Systems). Secondary antibodies conjugated with HRP were used for the western blot: goat anti-rabbit (ThermoFisher 31460) and goat anti-mouse (ThermoFisher 31430). Secondary antibodies (Goat IgG) conjudated with either Alexa 488, or 594 were used for immunostaining and they were from Invitrogen.
Validation	All the antibodies above are commonly used commercial antibodies.

Eukaryotic cell lines

Policy information about cell lines

Cell line source(s)	HEK293, HEK293FT, Neuro2a, H4, H9 human embryonic stem cell line, human iPSC line IMR90.
Authentication	HEK293, Neuro2a and H4 were directly purchased from ATCC. HEK293FT were directly purchased from ThermoFisher. H9 and IMR90 were directly purchased form WiCell.
Mycoplasma contamination	All the cells were tested and certified as mycoplasma-free.

Commonly misidentified lines (See ICLAC register)	None of the cell lines used is listed as commonly misidentified.

Animals and other organisms

Policy information about studies involving animals; ARRIVE guidelines recommended for reporting animal research

Laboratory animals	For neuron culture, hippocampal neurons were dissected from P0 CD-1 mice, either sex. For APP CRISPR viruses injection, 8-week old C57BL/6 mice (either sex) were used for hippocampi injection, and P0 C57BL/6 mice (either sex) were used for ICV injection.
Wild animals	The study did not involve wild animals.
Field-collected samples	The study did not involve field-collected sample.
